# Seasonal Fish Passage Patterns Through an Estuarine Fishway in Relation to Tidal Hydraulics

**DOI:** 10.3390/ani16193014

**Published:** 2026-09-24

**Authors:** Jianzhang Lv, Weijun Yang, Pengyu Zhu, Zhixiang Zheng, Biao Wang, Yongzeng Huang, Xiaogang Wang

**Affiliations:** 1 Hydraulic Engineering Department, Nanjing Hydraulic Research Institute, Nanjing 210029, China; jz_lv@tju.edu.cn (J.L.); biaowang@hhu.edu.cn (B.W.); yzhuang@nhri.cn (Y.H.); 2 State Key Laboratory of Hydraulic Engineering Intelligent Construction and Operation, Tianjin University, Tianjin 300350, China; 3 Cao’e River Basin Center, Shaoxing 312000, China; 4 Cao’e River Sluice Investment and Development Co., Ltd., Shaoxing 312000, China; zhulieyu12@yeah.net (P.Z.); 13626869023@163.com (Z.Z.)

**Keywords:** estuarine fishway, tidal hydraulics, seasonal fish passage, hydraulic opportunity window, imaging sonar, numerical simulation

## Abstract

Fish passage through estuarine fishways varies with tides and seasons, but their associations remain poorly understood. At the Cao’e River Sluice fishway, sonar monitoring quantified the timing and intensity of crossing events at a section near the freshwater-side exit over 961 half-hour intervals on 30 monitoring days, while numerical simulations characterized hydraulic conditions. Lower seaward levels reduced entrance depth, with local connectivity loss simulated at a seaward level of 0.20 m. At a seaward water level of 1.16 m, entrance depth averaged 0.55 m, and this screening reference was met for 5.7–6.2 h per 12 h cycle. Total passage intensities reached 371 and 344 ind/h during late flood and early ebb in March–June, compared with 43 and 57 ind/h during late ebb and subsequent early flood in September–November. Adjusted upstream intensity was lower in September–November than in March–June across tidal stages (ratios 0.03–0.18, *p* < 0.001), while daytime upstream intensity was 3.0–5.5 times that during 00:00–06:00 (*p* < 0.001). Elevated upstream crossing intensity at the exit section showed season-specific, non-monotonic associations with concurrent head difference (Δ*H*), occurring at Δ*H* = 2.25–3.75 m during rapid ebb in March–June and under small positive to reversed heads during flood or large positive heads during ebb in September–November. Mean target lengths in selected subsamples were 17.6 ± 3.4 and 26.8 ± 6.7 cm, respectively. Upstream movement predominated in both periods, and upstream intensity was somewhat higher during spring tides. These findings support maintaining entrance connectivity and considering seasonal passage timing and tidal conditions in fishway operation.

## Simple Summary

Estuarine fishways help fish move between coastal and freshwater habitats, but their effectiveness can change greatly as tides rise and fall. We studied the right-bank fishway at the Cao’e River Sluice using field monitoring and numerical simulations to examine tidal hydraulics and seasonal fish passage patterns. Simulations showed that lower seaward water levels reduced entrance depth, with hydraulic connectivity becoming restricted at very low levels. Fish passage was more active during March–June than during September–November, and movements from the seaward side toward the freshwater side predominated in both periods. Upstream passage peaks occurred around high tide during March–June and around low tide during September–November, indicating seasonal differences in passage timing relative to the tidal cycle. Fish passage was also generally more active during spring tides and daylight hours. These findings highlight the relevance of entrance connectivity and seasonal passage timing to estuarine fishway design and operation.

## 1. Introduction

Estuaries are ecological transition zones linking marine, estuarine, and freshwater habitats. Many fishes use different habitats across life-history stages for reproduction, recruitment, feeding, and overwintering, thereby sustaining functional connectivity among riverine, estuarine, and nearshore ecosystems [1]. Estuarine habitat function depends not only on local habitat quality but also on connectivity among habitats used at different life-history stages [2,3,4]. However, dams, weirs, sluices, and tidal-control structures in lower rivers and estuaries impede longitudinal fish movement and alter hydrological processes and habitat conditions, with persistent consequences for migratory and estuary-dependent fishes [5,6,7]. Restoring river connectivity has consequently become a major component of freshwater biodiversity conservation [8], and the recovery of fish assemblages after removal of tidal barriers demonstrates the direct role of estuarine connectivity in habitat use [9]. Where estuarine control structures must remain in place for flood protection, tidal exclusion, or water-resources management, fishways provide an important means of mitigating fragmentation and maintaining movement between marine and freshwater habitats.

Fishway construction alone does not ensure effective restoration of river connectivity; performance depends on whether fish can reach and locate the entrance, enter the facility, and ultimately complete passage. Early evaluations distinguished attraction, entry, and passage as sequential components of fishway effectiveness and showed that facility type, target species, monitoring method, and operating conditions all influence the outcome [10,11,12]. Subsequent studies have emphasized that fishway design must integrate ecological requirements, behavior, hydraulics, and facility operation rather than rely on a small set of fixed hydraulic criteria [13,14,15]. The defining feature of an estuarine fishway is the continuously varying seaward tidal boundary. When the freshwater-side level remains relatively stable, tidal fluctuations periodically alter the head difference and flow direction across the fishway, thereby changing entrance depth and submergence as well as the internal flow field [16,17]. These effects propagate through a sequence of hydraulic connectivity, entrance flow, and internal hydraulics. Seaward tidal level first determines whether a continuous water column is maintained at the entrance; the head difference then controls the direction and magnitude of entrance discharge; and the external boundary condition is translated along the fishway into changes in the water-surface profile, velocity, shear, and turbulence. Entrance flow should provide a detectable attraction cue while maintaining velocities that permit access by small-bodied or weak-swimming fish [18,19]. Once fish have entered, passage is governed not only by maximum and cross-section-averaged velocities but also by local hydraulic loading, the mainstream path, and the distribution of low-velocity and resting areas; species may respond differently to the same velocity and turbulence conditions [20,21,22]. Some fish can use appropriately scaled low-velocity zones and vortices for flow refuge and station holding or select mainstream margins and local low-velocity areas as passage routes [23,24]. An estuarine fishway therefore does not provide uniform passage conditions throughout a tidal cycle. Instead, synchronized changes in head difference, entrance submergence, and internal hydraulics may create stage-specific periods suitable for passage. Monitoring at tidal barriers likewise shows that fish passage is associated with tidal stage, flow direction, and facility operation [25,26,27].

Periodic hydraulic variation alone cannot explain passage through an estuarine fishway because the abundance, migratory motivation, and locomotor capacity of fish arriving near the facility also vary seasonally. Migration phenology in estuarine fishes is associated with life-history requirements such as reproduction, foraging, habitat transitions, and overwintering, and its timing is further modulated by environmental cues including temperature and water level [28,29]. A single migration season may contain multiple groups that differ in species, sex, life-history stage, and migration timing, with corresponding differences in upstream travel speed and estuarine residence time [30]. At shorter time scales, tidal stage, current direction, and tidal range can regulate the timing of fish activity. Acoustic monitoring at an estuary mouth revealed a close association between fish movements and tidal flow, while some individuals moved against the current, indicating that both tidal transport and active swimming contribute to short-term migration [31]. The use of tidal currents differs among species and life-history stages. Some fish exploit tidal flows for directed movement, whereas others alter activity timing, direction, or spatial position to avoid unfavorable conditions [32,33,34]. Body size, life-history stage, and water temperature also affect swimming speed, endurance, and sustainable passage distance and therefore determine the capacity to negotiate entrance flows, high-velocity zones, and resting areas [35]. Consequently, similar tidal and hydraulic conditions may elicit different passage responses among seasons because the migrating assemblages differ in motivation, species and size composition, swimming capacity, and behavioral strategy. Passage through an estuarine fishway thus fundamentally depends on temporal matching between periodically varying hydraulics and seasonally varying migration demand.

Current understanding of this matching remains limited at three levels. First, most studies of estuarine connectivity have focused on tide gates, culverts, and tidal barrages, whereas field studies of long, dedicated estuarine fishways are scarce; longitudinal hydraulic variation and the sustained passage process have therefore received insufficient attention. Second, fishway hydraulics are commonly evaluated under a small number of fixed water-level or discharge scenarios. Such analyses cannot represent continuous changes in head difference, entrance submergence, and internal hydraulics over an entire tidal cycle or identify how stage-specific favorable conditions develop and persist [36]. More importantly, fish monitoring and hydraulic analyses are often conducted independently. Monitoring studies typically describe statistical relationships between passage abundance and tidal level, discharge, or time, whereas hydraulic studies evaluate depth, velocity, and turbulence under selected operating conditions. A systematic understanding of the chain linking continuous tidal variation, hydraulic connectivity and internal hydraulics, and actual seasonal passage activity is still lacking. This gap constrains mechanistic interpretation and provides limited guidance for operational timing and hydraulic regulation.

This study investigated the right-bank estuarine fishway at the Cao’e River Sluice to examine three questions: (1) How do tidal-level changes affect hydraulic connectivity at the entrance, the water-surface profile, and the internal flow field? (2) How does fish passage differ among seasons across tidal and diel time scales? (3) How are upstream and downstream passage intensities associated with head difference, the rate of tidal-level change, and tidal category? The principal novelty lies in linking tidal variation, entrance connectivity, and internal flow conditions with seasonal fish passage performance in a long, dedicated estuarine fishway, for which field observations remain scarce. By integrating continuous tidal records, imaging-sonar monitoring, and three-dimensional hydrodynamic simulations, the study characterizes potential hydraulic passage windows and quantifies their correspondence with seasonal patterns of fish passage.

## 2. Materials and Methods

### 2.1. Study Area

The Cao’e River Sluice is located in the Cao’e River Estuary, Shaoxing, Zhejiang Province, China, and is an important tidal-control project on the southern bank of the Qiantang River Estuary. Its primary functions are flood and tidal protection, drainage, and water-resources development, with additional benefits for water-environment improvement and navigation. The sluice comprises 28 flood-discharge bays with a total clear width of 560 m, and the normal impoundment level upstream is 3.9 m. Fishways were installed on both banks to mitigate the effects of the project on longitudinal ecological connectivity. This study focused on the right-bank fishway located along the diversion dike on the right side of the sluice.

The right-bank fishway is approximately 482.5 m long and 2.0 m wide and comprises, from the freshwater side to the seaward side, the fishway exit section, straight section 1, a curved section, straight section 2, and the fishway entrance section. The mean bed slope of the pooled section is approximately 0.25%. The fishway uses alternating baffles spaced at 3.0 m, with a 6.0 m long resting pool every 27 m. Adjacent pools are connected by a notch and an orifice positioned on opposite sides of each baffle. Each notch is 1.0 m wide and 1.0 m deep and opens at the baffle crest. Each orifice is 0.8 m × 0.8 m and is located at the bed, providing passage routes through different parts of the water column. The notch becomes fully submerged at local water depths of 2.0 m or greater. The fishway entrance section is located on the seaward side in the Qiantang River Estuary, whereas the fishway exit section is located on the freshwater side upstream of the Cao’e River Sluice. During the monitoring periods, the gates at the seaward entrance and freshwater-side exit of the fishway remained fully open throughout each tidal cycle, with no adjustment according to tidal stage. The overall layout and principal structures are shown in Figure 1.

### 2.2. Monitoring Methods

The Cao’e River Sluice lies in a turbid estuarine environment characterized by high suspended-sediment concentrations and poor underwater visibility, which constrain conventional optical monitoring. Continuous fish-passage monitoring was therefore conducted using an Oculus M1200d multibeam imaging sonar (Blueprint Subsea, Ulverston, UK). The sonar operated at 1.2 MHz with horizontal and vertical fields of view of 130° and 20°, respectively. It was mounted on the sidewall of a pool near the fishway exit section, with the transducer elevation approximately level with the notch sill; the sonar sector covered both the notch and the orifice. Sonar recordings were manually reviewed. Passage direction was determined from continuous target trajectories, and individual targets were counted one by one. Movement from the seaward side toward the freshwater side was defined as upstream passage, whereas movement in the opposite direction was defined as downstream passage. Each continuous trajectory crossing the monitoring section was recorded as one passage event. Consecutive echoes belonging to the same target were linked across sonar frames, and temporally and spatially continuous trajectory segments were merged and counted once. Targets that approached the monitoring section without completing a crossing were excluded. Target identity was retained while the trajectory remained continuously visible within the sonar field; a subsequent crossing after loss of the trajectory was recorded as a new passage event. Passage counts therefore represent recorded crossing events at the monitoring section. Passage intensity is expressed in ind/h as a nominal measure of crossing frequency, with each recorded crossing contributing one count. Fish targets were distinguished from drifting debris and other non-fish echoes by examining target shape, trajectory continuity, and displacement across consecutive frames. Irregular echoes without a coherent body outline or continuous trajectory were excluded. Because imaging sonar has limited capacity for species identification, individual targets were not assigned to species.

Fish passage was monitored during May–June and September–November 2025 and during March and June 2026. The dataset comprised 30 monitoring dates, including 21 dates during March–June and nine dates during September–November. Because the monitoring duration differed among dates and tidal cycles, all subsequent statistical analyses used the exact effective monitoring duration. After unmonitored intervals were excluded, the analytical dataset comprised 961 observed 30 min intervals, equivalent to 480.5 h of effective monitoring. March–June included 529 intervals from 21 monitoring dates (264.5 h), whereas September–November included 432 intervals from nine monitoring dates (216 h). Observed zero counts were retained, while intervals without monitoring were excluded. Observations were therefore divided into these two seasonal groups for comparative analysis. For body-length analysis, clarity-selected samples comprised 200 targets from 5246 crossings on 18 June 2025 and 300 from 5347 crossings on 27 June 2026 (500 of 10,593 crossings; 4.7%). The September–November sample comprised 450 targets from 1204 crossings on 27 September 2025 and 50 from 185 crossings on 12 November 2025 (500 of 1389 crossings; 36.0%). Measurable targets had a complete, isolated and sufficiently straight body outline with visible head and caudal endpoints. Length was measured along the visible body axis in the clearest frame of each continuous trajectory using the scaled sonar image (Figure S1). Targets showing overlap, pronounced curvature or evident foreshortening were excluded, and each trajectory contributed one measurement. No numerical aspect-angle correction was applied. At 1.2 MHz, the M1200d has nominal range and angular resolutions of 2.5 mm and 0.6°, respectively. At the 2.0 m display range, the angular resolution corresponds to a maximum cross-range resolution of approximately 21 mm. Because field measurement accuracy was not independently calibrated, the values were treated as sonar-estimated apparent body lengths.

The sonar section was located near the freshwater-side fishway exit section. An upstream record therefore represents the time at which a fish crossed the monitoring section near the freshwater-side exit, whereas a downstream record represents movement from the freshwater side toward the fishway interior. Passage intensity was analyzed in relation to the tidal level and hydraulic conditions at the time each target crossed the sonar section. Fish moving upstream require time to travel from the seaward entrance to the exit on the freshwater side, so entry precedes the recorded completion of passage. The sonar analysis therefore quantified the temporal distribution of upstream crossings at the exit monitoring section and their correspondence with concurrent tidal conditions. The possible influence of this temporal offset was examined using assumed travel-time scenarios based on the simulated flow characteristics and plausible fish ground speeds.

### 2.3. Historical Biological Context for Fish Passage

Historical net-sampling records provided by the Cao’e River Basin Center were used to provide biological context for interpreting the seasonal passage patterns observed in the present sonar monitoring. In 2017, two net-sampling surveys were conducted each month from May to November near the freshwater-side exit of the right-bank fishway, for a total of 14 sampling events. The survey recorded 2943 aquatic organisms representing 27 taxa, including 24 fish species (Table S1). Because the sampling net was positioned near the fishway exit, the captured fishes largely represented individuals that had completed upstream passage through the right-bank fishway.

The original sampling records showed a pronounced seasonal shift in assemblage composition (Table 1). During May–June, which partially overlaps the March–June monitoring period considered in the present study, catches were numerically dominated by Japanese grenadier anchovy (*Coilia ectenes*) and sharpbelly (*Hemiculter leucisculus*), with 795 and 681 individuals, respectively. Their recorded body-length ranges were 10–27 and 8–19 cm, with corresponding body-weight ranges of 8–90 and 7–90 g. Small yellow croaker (*Larimichthys polyactis*) was also concentrated in this period, with 44 individuals and a body-length range of 3.5–7 cm. In contrast, September–November catches contained only 16 Japanese grenadier anchovy and no sharpbelly, while several medium- to large-bodied taxa became more prominent, including bighead carp (38 individuals; 15–60 cm), silver carp (30 individuals; 15–55 cm), crucian carp (27 individuals; 9–25 cm), topmouth culter (16 individuals; 13–38 cm), Mongolian culter (15 individuals; 14–30 cm), spotted seabass (15 individuals; 2.5–40 cm), and redlip mullet (8 individuals; 15–30 cm). These historical data indicate a transition from an assemblage numerically dominated by relatively small-bodied taxa in May–June toward a more heterogeneous assemblage containing several medium- to large-bodied taxa during September–November.

Swimming capacity also varies substantially among representative fishes potentially using the fishway. Japanese grenadier anchovy with a body length of 10–25 cm generally prefer flow velocities of 0.20–0.30 m/s, with a maximum tolerable velocity of approximately 0.40–0.50 m/s. Sharpbelly (*Hemiculter leucisculus*) with a mean body length of 11.6 ± 0.4 cm have a reported critical swimming speed of 0.593 ± 0.016 m/s [37], whereas Japanese eel elvers averaging 5.8 cm in total length have burst swimming speeds of 0.36–0.56 m/s and have difficulty progressing upstream against opposing velocities exceeding approximately 0.50 m/s [38]. These values indicate that juveniles and other relatively small-bodied fishes occurring during the spring–early summer migration period may be particularly sensitive to locally high velocities and may therefore benefit from periods of improved hydraulic connectivity and relatively favorable internal flow conditions.

Representative fishes occurring later in the year span a broader range of body sizes and include several taxa capable of tolerating higher flow velocities. Mullets with body lengths of 14–17 cm prefer velocities of approximately 0.40–0.60 m/s and have an upper velocity limit of approximately 0.80 m/s. Juvenile spotted seabass with a body length of 9.61 ± 0.74 cm have reported critical and burst swimming speeds of 0.69 ± 0.05 and 0.86 ± 0.05 m/s, respectively [39]. Absolute swimming speed generally increases with body length, such that larger individuals of a given species commonly attain higher absolute critical and burst swimming speeds than smaller conspecifics [35]. Accordingly, the occurrence of several medium- to large-bodied taxa during September–November suggests that the late-season fish assemblage may include individuals with a greater capacity to negotiate relatively high local velocities.

Historical capture surveys conducted in the same right-bank fishway in 2017 revealed clear seasonal differences in the dominant species composition and body-size characteristics of fishway users. Together with interspecific differences in swimming capacity, these historical field data provide an important biological basis for interpreting seasonal differences in fish-passage responses to tidal-hydraulic conditions in the present study.

### 2.4. Tidal-Level Dynamics

The seaward side of the Cao’e River Sluice is strongly influenced by tides in the Qiantang River Estuary and exhibits a pronounced semidiurnal signal, whereas the freshwater-side level remained close to 3.9 m during monitoring. To characterize typical tidal dynamics, continuous seaward tidal-level records from January 2025 to June 2026 were analyzed. One complete tidal cycle was defined from a low tide through the subsequent high tide to the next low tide. After anomalous and incomplete cycles were removed, flood and ebb portions of each complete cycle were normalized separately, mapped to a common relative tidal-time coordinate, and averaged at each time step to construct the mean tidal curve (Figure 2). The seaward tidal cycle was characterized by a rapid flood and a gradual ebb. Mean flood and ebb durations were approximately 3 and 9 h, respectively; mean high-tide level, low-tide level, and overall tidal level were 4.14, −1.34, and 1.16 m, respectively.

Tidal range varied among dates because of changes in the relative positions of the Moon and Sun and the lunar phase. Reinforcement of lunar and solar tidal forcing around the new and full moons generally produces spring tides, whereas partial cancellation around the first and third quarters produces neap tides; intermediate conditions were classified as moderate tides. Representative complete cycles were selected to illustrate typical tidal-level dynamics under neap, moderate, and spring tides.

Across the fish-passage monitoring dates, daily maximum tidal levels ranged from 2.85 to 5.57 m and daily minimum levels from −1.90 to −0.80 m. The observations thus encompassed a broad range of tidal levels and tidal ranges, providing a basis for evaluating fishway hydraulics and passage responses under different tidal conditions.

### 2.5. Numerical Simulation

A three-dimensional hydrodynamic model of the right-bank fishway was developed in FLOW-3D to examine internal hydraulics under varying tidal levels. The renormalization-group (RNG) *k*–*ε* turbulence model was used. Its strain dependent formulation is suitable for representing the rapidly strained, separated, and recirculating flows generated around the fishway baffles, notches, and orifices. The volume of fluid (VOF) method was used to track the free surface. Water-level boundary conditions were prescribed at both ends. The freshwater-side level was fixed at 3.90 m in accordance with sluice operation, whereas the seaward boundary was assigned the level for each scenario to represent positive and reverse head conditions generated by the tide. The bed, sidewalls, and baffles were treated as no-slip solid boundaries with a Manning roughness coefficient of 0.025. The flow field was considered stable when water levels and boundary discharges varied by less than 0.5% over five consecutive output intervals and the relative imbalance between inflow and outflow discharges was below 1%. A structured mesh was used, with local refinement around the notches, orifices, pools, and zones of rapid flow variation. Grid independence was assessed using meshes containing approximately 7 million, 12 million, and 16 million cells, and the comparison of longitudinal water-surface profiles is presented in Figure S2. Based on this assessment, the medium-density mesh containing approximately 12 million cells was adopted for the simulations. The time step was adjusted adaptively according to flow stability, and results were extracted after the water-surface profile and principal flow structures had stabilized in each scenario.

A local Cartesian coordinate system was established to standardize spatial descriptions. The upstream boundary of the fishway exit section was defined as *x* = 0; the *x*-axis extended generally along straight section 1 toward the fishway entrance section; the centerline section of straight section 1 corresponded to *y* = 0; and *z* denoted elevation. Because the curved section prevents planar coordinates from representing longitudinal position unambiguously, a streamwise distance *S* was also defined along the fishway centerline, beginning at the freshwater-side centerline origin and increasing toward the fishway entrance section. The *S* ranges for the fishway exit section, straight section 1, curved section, straight section 2, and fishway entrance section were 0–48.2, 48.2–120.4, 120.4–232.6, 232.6–431.5, and 431.5–482.5 m, respectively.

Model performance was evaluated in a representative regular pool at *x* = 56 m in straight section 1 under a seaward tidal level of 4.14 m. Surface velocities were measured with an acoustic Doppler current profiler (ADCP) at 16 evenly distributed points (Figure S3) and compared with simulated values at corresponding locations. Agreement was strong (Figure 3), with a coefficient of determination of *R*^2^ = 0.96 and a root-mean-square error (RMSE) of 0.03 m/s. The model showed good agreement with the measured surface velocities under this validation condition.

Based on the representative tidal cycle described in Section 2.4, the freshwater-side level was held at 3.90 m and the seaward level was set to 4.14, 3.80, 2.97, 1.16, and 0.20 m to cover representative conditions from high to low tide. The head difference was defined as Δ*H* = *H_f_* − *H_s_*, where *H_f_* and *H_s_* are the freshwater-side and seaward water levels, respectively. The five scenarios therefore corresponded to Δ*H* values of −0.24, 0.10, 0.93, 2.74, and 3.70 m, representing reverse head, small positive head, moderate positive head, large positive head, and a low seaward water level under a very large positive head, respectively. The scenarios are summarized in Table 2. Hydraulic characteristics were analyzed at the whole-fishway and representative-section scales. At the whole-fishway scale, water-surface elevation and depth were extracted along the centerline for all five scenarios to compare water-surface profiles and depth responses to seaward tidal level. At the section scale, a freshwater-side section (*x* = 50–80 m; *S* = 69.3–99.3 m) and a seaward section (*x* = 355–385 m; *S* = 378.0–408.0 m) were selected. Each 30 m section contained one resting pool and its adjacent upstream and downstream pools. Horizontal velocity fields were extracted at *z* = 3.1 and 2.1 m in the freshwater-side section and at *z* = 2.1 and 1.1 m in the seaward section to compare flow distributions within regular pools and resting pools and near the notches and orifices.

For the duty-cycle assessment, a 0.50 m entrance depth was adopted as a conservative operational screening criterion, considering the body-size range of representative fishes summarized in Section 2.3 and the requirement for a continuous water column at the entrance. The duration corresponding to this criterion was calculated from the 5 min tidal records for the 30 monitoring dates and expressed as hours and percentages of a standardized 12 h tidal cycle.

### 2.6. Statistical Analysis

Upstream and downstream passage counts were analyzed separately using negative binomial generalized linear mixed models at a temporal resolution of 30 min. This temporal resolution was consistent with that of the original passage records and allowed each observation to be matched directly with the corresponding tidal stage and hydraulic conditions. The natural logarithm of the actual monitoring duration of each observation was included as an offset, and monitoring date was specified as a random intercept. Fixed effects included monitoring period, tidal stage, tidal category, diel period, head difference (Δ*H*), tidal-level change rate (*dZ*/*dt*), and daily mean air temperature. Because concurrent water-temperature records were unavailable, daily mean air temperature at 2 m above the land surface for the Shaoxing area (30.00° N, 120.60° E), obtained from the ERA5-Land reanalysis dataset, was included as an approximate environmental covariate. The interaction between monitoring period and tidal stage was included to account for differences in stage-specific passage patterns between the two monitoring periods. Continuous covariates were standardized before model fitting. For presentation, the estimated coefficients and their confidence limits were converted from the standardized scale to the original measurement units using standard deviations of 1.984 m for Δ*H*, 1.469 m/h for *dZ*/*dt*, and 4.743 °C for daily mean air temperature. The adjusted passage-intensity ratios for these variables therefore represent multiplicative changes in predicted passage intensity associated with increases of 1 m, 1 m/h, and 1 °C, respectively. Pairwise Pearson correlations were calculated among the continuous variables and the binary-coded monitoring-period and tidal-stage terms. Within-date temporal dependence was evaluated using lag-one conditional Pearson residual correlations. As a sensitivity analysis, complete adjacent observations were aggregated into 1 h blocks by summing passage counts and monitoring durations and averaging the continuous hydraulic variables. Tidal stage and diel period were assigned according to the midpoint of each 1 h block, and the same model structure was refitted. The negative-binomial variance was parameterized as Var(*Y*) = *μ* + *μ*^2^/*θ*. Models were fitted using a custom maximum-likelihood routine in Python 3.12.14. Parameter estimation used adaptive Gauss–Hermite quadrature with 15 nodes, and numerical stability was evaluated using 7 and 11 nodes. Body-length distributions were summarized using the median and 10th and 90th percentiles and were compared between monitoring periods using a two-sided Mann–Whitney *U* test, with statistical significance evaluated at *p* < 0.05.

## 3. Results

### 3.1. Fishway Hydraulics Under Different Tidal Levels

Water-surface profiles and depths along the fishway under different seaward tidal levels are shown in Figure 4. Hydraulic characteristics for the five scenarios are summarized in Table S2. Under the reverse head at 4.14 m, the water surface rose gradually toward the sea; at 3.80 m, the profile was nearly horizontal. When the seaward level decreased to 2.97 m, a distinct drawdown profile developed in straight section 2. The longitudinal water-surface drop increased further at 1.16 and 0.20 m. Across the full length of straight section 2 (*S* = 232.6–431.5 m), water-surface drops were approximately 2.23 and 2.57 m, corresponding to mean surface slopes of approximately 1.12% and 1.29%, respectively. Thus, seaward tidal level substantially altered the overall water-surface profile: backwater effects produced a relatively flat profile at high tide, whereas lower tides increased the longitudinal drop and reduced depth in the seaward portion of the fishway.

The effect of seaward tidal level on water depth was spatially nonuniform. Depth varied little in the freshwater-side fishway exit section, straight section 1, and the curved section, whereas most depth reduction occurred from the downstream end of straight section 2 to the fishway entrance section. As the seaward level fell, the water-surface slope increased and depth declined rapidly in this zone, which progressively became the principal hydraulic constraint on passage. Even at a seaward level of 1.16 m, minimum depths in the fishway exit section, straight section 1, and curved section remained approximately 2.60, 2.49, and 2.53 m, respectively. Depth limitation was concentrated near the fishway entrance section (*S* ≈ 413–463 m). At seaward levels of 4.14, 3.80, and 2.97 m, depths in this zone were 3.25–3.59, 2.92–3.25, and 2.09–2.42 m, respectively. At a seaward tidal level of 1.16 m, entrance depth decreased to 0.51–0.61 m, with a mean of approximately 0.55 m, while hydraulic connectivity was retained. The 0.50 m screening criterion was selected with reference to the lower end of the A4 entrance-depth range and the need to maintain a continuous water column at the entrance. Under the lowest simulated seaward level of 0.20 m, the water column at the entrance became locally discontinuous, indicating an effective loss of hydraulic connectivity.

Using 1.16 m as the seaward-level reference associated with the 0.50 m screening criterion, this reference level was met or exceeded for approximately half of each standardized 12 h tidal cycle on the monitoring dates (Table S3). During March–June, the mean duration was 5.78 h under spring tides and 5.66 h under moderate–neap tides, accounting for 48.2% and 47.2% of the cycle, respectively. During September–November, the corresponding durations were 6.17 and 6.08 h, accounting for 51.4% and 50.7% of the cycle, respectively. The corresponding screened periods coincided with 71.5% of upstream crossings recorded near the freshwater-side exit during March–June and 33.9% during September–November.

Velocity fields at the elevations of the notches are shown in Figure 5, with corresponding contour lines provided in Figure S4. The alternating openings caused the mainstream to turn repeatedly between adjacent pools, producing an S-shaped flow path. Low-velocity recirculation zones developed along pool margins and near baffles, while the mainstream expanded and decelerated in resting pools. The freshwater-side section remained relatively stable, whereas the seaward section was more responsive to tidal level. In the freshwater-side section (*x* = 50–80 m, *z* = 3.1 m), flow was directed from the seaward side toward the freshwater side at a seaward level of 4.14 m (Δ*H* = −0.24 m), with mainstream velocities of approximately 0.3–0.7 m/s. At 3.80 m (Δ*H* = 0.10 m), velocities generally decreased to 0.1–0.3 m/s. When the seaward level fell to 2.97 and 1.16 m (Δ*H* = 0.93 and 2.74 m), the mainstream reversed toward the sea and velocities increased to approximately 0.4–0.6 and 0.5–0.7 m/s, respectively; recirculation-zone velocities were mostly below 0.3 m/s. Low-velocity areas occupied approximately half of the resting pool at *x* = 62–68 m, providing refuge and recovery space for weaker-swimming pelagic and mid-water fishes. In the seaward section (*x* = 355–385 m, *z* = 2.1 m), most velocities were below 0.3 m/s at seaward levels of 4.14 and 3.80 m. At 2.97 m, a continuous mainstream of approximately 0.4–0.6 m/s developed. At 1.16 m, reduced local depth and a steeper water-surface slope increased mainstream velocity to 0.4–0.7 m/s, with local values of 0.8–1.0 m/s near *x* ≈ 360–366 m, approaching the critical swimming speeds of some pelagic and mid-water fishes. More than 80% of the resting pool at *x* = 367–373 m remained below 0.3 m/s at the notch elevation, preserving its energy-dissipation and resting function. Low-velocity routes along mainstream margins and within recirculation zones could facilitate stepwise passage by weak swimmers.

At the elevation of the orifices, flow turned successively through the alternating openings and formed an S-shaped mainstream, accompanied by low-velocity recirculation zones in regular pools and a more concentrated low-velocity area in each resting pool (Figure 6; corresponding contour lines in Figure S5). The freshwater-side section (*x* = 50–80 m, *z* = 2.1 m) was relatively insensitive to tidal level. Most velocities were below 0.3 m/s at seaward levels of 4.14 and 3.80 m; when the level fell to 2.97 and 1.16 m, mainstream velocities between orifices increased to 0.4–0.7 m/s. The seaward section (*x* = 355–385 m, *z* = 1.1 m) was more sensitive. As the seaward level decreased from 2.97 to 1.16 m, mainstream velocities increased from 0.4–0.7 to 0.7–1.0 m/s, with local values exceeding 1.0 m/s. The continuous distribution of these high-velocity zones formed a potential velocity barrier for small-bodied or weak-swimming benthic fishes. Resting pools and recirculation zones nevertheless retained local low-velocity routes and recovery space.

The swimming characteristics summarized in Section 2.3 indicate preferred flow velocities of approximately 0.20 to 0.60 m/s among representative fishes, with reported critical and burst swimming speeds spanning approximately 0.36 to 0.86 m/s [37,38,39]. In the simulated flow fields, mainstream velocities under positive head conditions commonly reached 0.4 to 0.7 m/s, while pool margins and resting areas retained velocities below 0.3 m/s. Upstream progression may therefore involve alternating movement through faster flow and use of slower water. Opposing currents reduce ground speed during active swimming, while repeated turns and residence within pools further influence the mean rate of progression. Considering these biological and hydraulic characteristics, effective mean ground speeds of 0.05 to 0.3 m/s were assumed for the scenario analysis. The magnitude of this range is supported by field observations of fish movement at estuarine passage structures [25,40]. To account for repeated lateral turns along the mainstream, an effective travel distance of approximately 530 m was assumed, representing an increase of about 10% over the fishway centerline length of 482.5 m. These assumptions yielded travel-time scenarios of approximately 0.5 to 3 h for examining the possible temporal offset between seaward entry and exit-section detection.

### 3.2. Diel Variation in Passage Abundance on Representative Dates

Diel variation in passage abundance on representative dates is shown in Figure 7. Patterns were broadly consistent between the two representative dates in March–June. Upstream passage occurred throughout the day, with targets recorded in every 30 min monitoring interval, and abundance fluctuated continuously with multiple peaks. On 18 June 2025, the principal sustained peak occurred from 08:45 to 12:45 and accounted for 62.2% of the daily upstream total; the maximum passage count in a single 30 min interval was 739, with intermittent secondary peaks thereafter. Multiple upstream peaks also occurred on 27 June 2026, when the maximum 30 min passage count was 337 and passage counts of 149–168 occurred repeatedly. On both dates, high upstream counts occurred during the flood tide and remained relatively high into the subsequent ebb tide, indicating sustained upstream activity during March–June.

Passage during September–November was also dominated by upstream movement, but diel abundance was lower and alternated between sustained low counts and brief increases, yielding more discrete peaks. On 27 September 2025, upstream targets were recorded in 85.4% of the 30 min intervals. The principal peak from 08:15 to 11:45 accounted for 56.4% of the daily upstream total, and the maximum 30 min passage count was 122. On 12 November 2025, upstream targets occurred in only 52.1% of the intervals; the maximum passage count was 23, and peak duration was short. On both dates, the principal peaks occurred during the ebb tide as water level approached low tide, with only local secondary peaks during the flood tide.

Among the clarity-selected targets, apparent body length averaged 17.6 ± 3.4 cm for the two June samples and 26.8 ± 6.7 cm for the September and November samples. The corresponding medians were 17.6 and 26.8 cm, with 10th–90th percentile ranges of 13.2–22.0 and 18.3–35.3 cm, respectively. The length distributions differed significantly between these selected samples (Mann–Whitney *U* = 32,633, *p* < 0.001; Figure S6). Overall, the tidal-phase distributions of upstream passage differed between periods. During March–June, upstream activity persisted for longer, and high values extended from the flood tide into the subsequent ebb-tide period. During September–November, upstream activity was more intermittent, and high values were concentrated mainly in the late ebb tide and around low tide.

### 3.3. Tidal-Stage Distribution and Diel Rhythm of Passage Intensity

Sonar records from 30 monitoring dates in 2025–2026 were used to analyze passage intensity by tidal stage and diel period. To standardize tidal cycles among dates, the transition from low tide to flood tide was defined as relative time *t* = 0, and each complete low tide–high tide–subsequent low tide cycle was normalized to 12 h. The midpoint of each monitoring interval was used as its timestamp. Actual flood tides were mapped linearly to 0–3 h and ebb tides to 3–12 h, after which passage records were assigned to tidal stages. Each flood and ebb period was divided into equal early and late halves: early flood tide (stage 1, 0–1.5 h), late flood tide (stage 2, 1.5–3.0 h), early ebb tide (stage 3, 3.0–7.5 h), and late ebb tide (stage 4, 7.5–12.0 h) (Figure 8a). Passage intensity for each stage was calculated as *I* = *N*/*T*, where *N* is the cumulative number of recorded crossing events and *T* is the actual monitoring duration (h); *I* is expressed in ind/h. Upstream records represented crossings of the monitoring section near the freshwater-side exit, which could occur at a different tidal stage from entry, whereas downstream records represented crossings in the opposite direction, from the freshwater side toward the fishway interior. During March–June, 47,600 upstream and 19,205 downstream crossings were recorded, yielding 66,805 total crossings and a net upstream count of 28,395. Downstream crossings accounted for 28.7% of the total. During September–November, 5187 upstream and 1177 downstream crossings were recorded, yielding 6364 total crossings and a net upstream count of 4010; downstream crossings accounted for 18.5%.

During March–June, passage intensity increased from the early to the late flood tide, remained high during the early ebb tide, and decreased during the late ebb tide (Figure 8b). Total intensities across the four stages were 207, 371, 344, and 130 ind/h, respectively. The maximum occurred during the late flood tide, whereas the early-ebb value was only 7.2% lower; together, these stages formed the principal high-intensity period around the transition at high tide. Upstream intensities followed the same pattern at 136, 281, 254, and 78 ind/h. Downstream intensity varied less among stages, with values of 91 and 90 ind/h during the late flood and early ebb tides, respectively. The upstream proportion peaked at 75.6% during the late flood tide, was 73.8% during the early ebb tide, and declined to 59.9% during the late ebb tide. These patterns indicated a sustained period of high upstream passage intensity around high tide, extending from the late flood to the early ebb tide.

A different stage distribution occurred during September–November. Passage intensity was highest during the early flood tide, then decreased to a minimum during the early ebb tide before increasing again during the late ebb tide (Figure 8b). Total intensities across the four stages were 57, 29, 8, and 43 ind/h, respectively; upstream intensities were 47, 21, 6, and 36 ind/h, and downstream intensities were 10, 8, 2, and 7 ind/h. Both directions exhibited similar stage patterns, with upstream passage predominating throughout and accounting for 83.0% and 84.7% during the early flood and late ebb tides, respectively. Upstream crossings recorded near the freshwater-side exit were therefore concentrated around low tide, linking the late ebb tide to the early flood tide of the subsequent cycle.

Diel rhythm was analyzed in Beijing time without phase-shifting records by tide. Passage records from each monitoring date were assigned to 24 hourly intervals, and intensity was calculated as cumulative abundance divided by cumulative monitoring duration within each interval. Passage in both periods was concentrated during daylight, although peak timing differed (Figure 9). March–June exhibited a morning primary peak and an evening secondary peak. Intensity increased rapidly after 05:00, and the main high-intensity period occurred from 06:00 to 10:00; total intensity peaked at 579 ind/h between 09:00 and 10:00. Activity increased again from 16:00 to 18:00 and reached 472 ind/h between 16:00 and 17:00. When summarized in 6 h blocks, mean total intensities were 420 and 335 ind/h during 06:00–12:00 and 12:00–18:00, respectively, both exceeding those in the remaining periods. During September–November, high values were concentrated from 08:00 to 13:00. Intensity peaked at 117 ind/h between 09:00 and 10:00 and remained at 95–99 ind/h from 10:00 to 13:00; it declined after 14:00, with no distinct evening peak. Diel peaks in both periods were driven primarily by upstream movement, indicating a shared pattern of enhanced daylight activity, particularly in the morning, together with a secondary evening peak during March–June. Of the 47,600 upstream crossings recorded during March–June, 12,031 (25.3%) occurred on 20 June 2025. When this date was excluded, the highest pooled hourly total passage intensity shifted from 09:00–10:00 (579 ind/h in the full dataset) to 16:00–17:00 (472 ind/h), while the intensity at 09:00–10:00 decreased to 330 ind/h, indicating that the prominence of the morning maximum was sensitive to this high-count date.

### 3.4. Responses of Passage Intensity to Head Difference, Rate of Tidal-Level Change, and Tidal Category

To further examine the relationship between passage intensity and tidal dynamics, upstream passage intensity was mapped in a two-dimensional space defined by head difference and the rate of tidal-level change. The distribution was constructed by matching sonar passage records with the water level series at the corresponding observation times. Passage intensity was calculated within hydraulic bins, and bins without observations were filled using inverse distance weighting. Regional means were calculated as arithmetic averages of the gridded intensities within the specified hydraulic ranges, including interpolated cells. Head difference was defined as the freshwater-side level minus the seaward tidal level; Δ*H* > 0 indicates a higher water level on the freshwater side. The tidal-level change rate, *dZ*/*dt*, was defined as the temporal rate of change in the seaward tidal level, with positive values indicating the flood tide and negative values indicating the ebb tide. Both seasonal periods exhibited clustered high-intensity regions, but the corresponding hydraulic conditions differed.

During March–June, high upstream intensities occurred mainly under positive heads during both flood and ebb tides (Figure 10a). The principal region occurred during rapid ebb at rates of −1.5 to −1.1 m/h and Δ*H* = 2.25–3.75 m, with a regional mean of approximately 548 ind/h and a maximum of 1252 ind/h at Δ*H* = 2.75 m and a rate of −1.1 m/h. Records from 20 June 2025 contributed substantially to the maximum cell value of 1252 ind/h, indicating that the magnitude of this local maximum was sensitive to the inclusion of this high-count date. During slower ebb at −0.9 to −0.3 m/h, high values occurred at Δ*H* = 0.25–1.25 m, with a regional mean of 276 ind/h and a maximum of 624 ind/h. Elevated intensities also occurred within this head range during flood tide at rates of 0.5–1.1 m/h, averaging 281 ind/h. Intensity generally declined when positive head exceeded 3.75 m.

During September–November, upstream intensity was lower, with two principal high-value regions (Figure 10b). During flood tide, elevated intensities occurred at rates of 0.9–1.3 m/h and Δ*H* = −1.25–0.75 m, with a regional mean of approximately 54 ind/h and a maximum of 213 ind/h at Δ*H* = −0.75 m and a rate of 1.1 m/h. During ebb tide, a relatively continuous region occurred at rates of −0.9 to −0.3 m/h and Δ*H* = 4.75–5.75 m, averaging 41 ind/h and reaching 89 ind/h. These elevated upstream crossing intensities recorded near the freshwater-side exit under large positive heads corresponded to a concentration of exit-section detections near low tide.

Table 3 shows that the relationship between tidal category and upstream passage intensity varied between monitoring periods and among tidal stages. During March–June, differences between tidal categories were relatively moderate. Passage intensity under spring tides was 1.35, 1.14, and 1.55 times that under moderate–neap tides during the early flood, early ebb, and late ebb stages, respectively, whereas the ratio was 0.79 during the late flood stage. During September–November, passage intensity was higher under spring tides during all four stages, with the largest differences occurring during the early flood and late ebb stages. The respective intensities under spring and moderate–neap tides were 158 and 10 ind/h during the early flood stage and 70 and 16 ind/h during the late ebb stage, corresponding to ratios of 16.45 and 4.39. The ratios during the late flood and early ebb stages were 1.69 and 1.05, respectively.

### 3.5. Model-Based Analysis of Passage Intensity

The models were based on 961 valid observations at a temporal resolution of 30 min, representing 480.5 h of monitoring across 30 dates. After accounting for monitoring effort, tidal and hydraulic conditions, daily mean air temperature, and date-level variation, the results revealed clear temporal structuring of fish passage intensity (Table 4). Monitoring period showed the most consistent association: across the four tidal stages, adjusted passage intensity during September–November was 0.033–0.177 times that during March–June for upstream passage and 0.024–0.102 times that during March–June for downstream passage, with all stage-specific differences having *p* values below 0.001.

Tidal-stage patterns differed between the two monitoring periods. During March–June, passage intensity increased from the early flood to the late flood stage and decreased during the late ebb stage. During September–November, passage intensity during the early and late ebb stages was lower than that during the early flood stage. Diel period was also significantly associated with both passage directions, with the highest adjusted upstream passage intensity occurring during 06:00–12:00.

Evidence for associations with tidal category and daily mean air temperature was stronger in the upstream model. Adjusted upstream passage intensity under spring tides was 1.702 times that under moderate–neap tides (95% CI: 1.010–2.866, *p* = 0.046), and each increase of 1 °C in daily mean air temperature corresponded to an adjusted ratio of 1.135 (95% CI: 1.073–1.202, *p* < 0.001). The corresponding downstream confidence intervals included 1. Across the observed hydraulic range, the estimated average linear associations of Δ*H* and *dZ*/*dt* with passage intensity were modest in both directions, with adjusted ratios ranging from 0.930 to 1.074 and confidence intervals including 1. The correlation between monitoring period and daily mean air temperature was −0.185, while the largest absolute correlation among the examined terms was 0.781 between the late-ebb indicator and Δ*H* (Table S4).

The 1 h sensitivity analysis included 473 complete blocks from the same 30 monitoring dates. The stage-specific ratios for September–November relative to March–June remained below 1 in both passage directions, and the positive upstream association with daily mean air temperature was retained. At the 1 h resolution, the adjusted upstream ratio for spring tides relative to moderate–neap tides was 1.681 (95% CI: 0.993–2.845, *p* = 0.053). The negative-binomial dispersion parameters *θ* were 0.682 and 1.107 for the upstream and downstream models, and the corresponding conditional Pearson dispersion ratios were 1.147 and 1.276. Lag-one residual correlations were 0.502 and 0.454 at the 30 min resolution and 0.386 and 0.416 at the 1 h resolution, respectively. Complete sensitivity results are presented in Table S5.

## 4. Discussion

### 4.1. Biological Basis of Seasonal Differences in Fish Passage

Estuarine fishways connect marine, estuarine, and freshwater habitats, and fish move seasonally in response to reproduction, foraging, recruitment, and overwintering requirements. Passage intensity is therefore jointly influenced by abundance, species composition, body size, and migratory motivation. Previous studies have documented interspecific and size-related differences in passage abundance and timing [41,42]. The two seasonal periods differed in passage intensity, peak timing, and the body size of sonar-detected targets. The selected target-length subsamples differed between the two sets of dates, with mean lengths of 17.6 ± 3.4 cm for the two June samples and 26.8 ± 6.7 cm for the September and November samples. Historical surveys documented seasonal variation in assemblage composition and body-size structure in 2017 and provide context for the present observations.

Historical surveys conducted in May–June, which partially overlaps the March–June monitoring period, recorded catches dominated by Japanese grenadier anchovy and sharpbelly, with small yellow croaker also present (Table S1). Japanese grenadier anchovy are anadromous and migrate from coastal waters into estuaries and rivers to reproduce [43]. This assemblage composition provides biological context for the relatively high passage intensity observed during March–June. Although larval and juvenile estuarine fishes have limited swimming capacity, they can selectively exploit tidal currents for directed movement by adjusting their position in the water column and the timing of activity. This behavior is consistent with selective tidal-stream transport, whereby fishes and other aquatic animals adjust activity, vertical position, and movement direction to migrate or retain position in favorable habitats [44]. Recent studies further show that entry and retention of early-life-stage fishes in estuaries result from interactions between behavioral regulation and tidal transport; stage-specific vertical-position selection can facilitate access to nursery habitats or reduce seaward export [45,46]. In the present study, exit-section crossing intensity increased during the late flood tide and remained high into the early ebb tide. Mean target length in the selected June samples was 17.6 ± 3.4 cm. This timing may reflect the combined influence of tidal transport and continued active upstream movement across the high-tide transition.

Mean body length measured from the selected sonar images was greater during September–November, reaching 26.8 ± 6.7 cm. Historical catches during this period included bighead carp, silver carp, crucian carp, topmouth culter, Mongolian culter, and spotted seabass among the more abundant taxa, while redlip mullet and common carp were represented by eight and two individuals, respectively (Table S1). This assemblage provides context for considering a range of seasonal movements. Estuarine habitats support feeding, juvenile growth, and recovery of body condition [47,48]. Studies of seabass and grey mullets describe movements associated with feeding and seasonal habitat use across coastal and estuarine waters [49,50,51]. Research on common carp and silver carp also illustrates movements among connected freshwater habitats associated with foraging, overwintering, and changing habitat conditions [52,53]. These studies provide broader ecological context for possible movement motivations within the assemblage recorded in the historical surveys. These life-history processes provide possible context for the more episodic passage pattern observed during September–November. Upstream movement accounted for 84.7% and 83.0% of passage during the late ebb and early flood tides, respectively, indicating that exit-section crossings were concentrated around low tide. Targets selected from the September and November recordings were larger on average than those selected from the two June recordings.

Diel patterns differed between monitoring periods. Both periods showed morning peaks, while March–June also exhibited a distinct evening peak. Comparable monitoring has revealed species-specific diel patterns: passage through a tidal-exclusion barrier varied with diel period and movement direction [40]; salmon and sea trout at a barrier in the Rhine–Meuse Estuary passed mainly during daylight, whereas lamprey showed no clear diel preference [54]; and barbels in a Po River fishway passed preferentially at night, a pattern not shared by other fishes [42]. Species composition, foraging activity, and movement motivation provide possible biological contexts for the observed temporal patterns.

Overall, March–June was characterized by higher and more sustained passage intensity, whereas September–November showed lower and more episodic passage activity. The selected target-length subsamples differed between the two sets of dates, and historical fishway surveys documented seasonal variation in assemblage composition. Together, these observations provide a basis for hypotheses concerning biological contributions to the seasonal passage patterns.

### 4.2. Tidal-Hydraulic Associations with Fish Passage

An estuarine fishway is controlled simultaneously by a relatively stable freshwater-side level and a periodically varying seaward tidal level. Tidal variation alters entrance submergence, head difference, and internal hydraulics, creating potential passage windows over the tidal cycle [16,54]. In this study, tidal effects were concentrated from the seaward end of straight section 2 to the entrance. Simulated entrance depth averaged approximately 0.55 m at a seaward level of 1.16 m, whereas hydraulic connectivity was effectively lost locally at 0.20 m. Entrance depth and the duration for which the adopted screening condition was met were used to characterize a potential hydraulic window at the entrance.

Once hydraulic connectivity is maintained, flow must support both entrance detection and sustained passage. Moderate positive discharge can provide an attraction cue, while an excessive head may increase the energetic cost of upstream movement. Addicoat et al. [40] and Meijer zu Schlochtern et al. [54] reported passage through tidal barriers near water-level equilibrium and at relatively low velocities. In the present fishway, elevated intensities of upstream crossings recorded near the freshwater-side exit were also concurrent with larger positive heads, and the simulated flow fields showed a distinct mainstream bordered by low-velocity areas. At a seaward level of 3.80 m, mainstream velocities of 0.1 to 0.3 m/s included the preferred range of Japanese grenadier anchovy. At 2.97 m, velocities of 0.4 to 0.6 m/s overlapped the preferred range of mullets, the critical swimming speed of sharpbelly, and the burst swimming range of Japanese eel elvers. At 1.16 m, local velocities reached 0.8 to 1.0 m/s at the notch elevation and exceeded 1.0 m/s near the bottom, reaching or exceeding several swimming-capacity values summarized in Section 2.3. Passage suitability therefore depends on both the extent of high velocity zones and access to slower routes and resting areas [55,56,57,58,59,60]. Modelling that incorporates resting behavior and energy expenditure further highlights the importance of recovery during passage through long fishways [61].

Whether a potential hydraulic window produces actual passage further depends on matching flow conditions to life-history demand and swimming capacity. Seasonal differences in target size may modify how fish respond to the changing hydraulic conditions. The smaller targets recorded during March–June may have been more sensitive to reduced entrance depth and relatively high local velocities, consistent with the concentration of high passage intensity around high tide. In contrast, the larger targets recorded during September–November may have had a broader capacity to negotiate relatively strong flows, potentially contributing to the different timing of upstream crossings recorded near the freshwater-side exit. Hydraulic barriers exert selective effects among species and body sizes [62], and fish may use prolonged or sprint swimming to traverse flow zones of different lengths and difficulty [63]. Passage was more active under spring tides than under moderate–neap tides in both periods, but enhancement was concentrated in different tidal stages. These observations indicate that tidal category was associated with differences in the timing and duration of elevated passage intensity.

Upstream crossings recorded near the freshwater-side exit may occur after a period of movement and residence within the fishway, so the concurrent hydraulic conditions may differ from those encountered at the seaward entrance. During September–November, 55.2% of upstream crossings occurred during late ebb, and this stage remained dominant after detection times were shifted backward by 0.5–3 h, accounting for 60.6–70.6% of crossings (Table S6). Thus, the late-ebb concentration persisted across the tested lag scenarios, although only 33.9% of autumn crossings coincided with the adopted depth-screened period at detection. Earlier entry followed by prolonged residence within the fishway offers a possible explanation for this temporal mismatch. Uncertainty in simulated entrance drying and local movements of fish residing in the upper pools may also contribute, as entrance wetting and drying and individual movement histories were not observed. The low overlap therefore does not demonstrate entry through a disconnected entrance. At a finer hydraulic scale, the March–June Δ*H*–*dZ*/*dt* distributions showed little spatial similarity to the zero-lag distribution at assumed lags of 2.5–3 h (Table S7), illustrating the sensitivity of the mapped hydraulic associations to the assumed transit time. Figure 10 characterizes hydraulic conditions concurrent with exit-section crossings; actual entry conditions during September–November remain unknown. Synchronized entrance and exit monitoring with individual tracking would help clarify the relationship between entry conditions, residence within the fishway, and exit timing.

### 4.3. Operational Implications and Future Research

Effective passage through an estuarine fishway depends on the temporal matching of hydraulic opportunities with seasonal fish movement. Temperature, salinity, and dissolved oxygen may further influence fish aggregation and passage motivation [16,40,54], while interactions among body size, temperature, and velocity affect swimming performance [35]. These relationships support joint consideration of hydraulic, biological, and environmental conditions in fishway operation.

Engineering design and operation should account for the duration and continuity of hydraulic windows and their compatibility with the capacities of target fishes. Design scenarios should span tidal categories, upstream–downstream water-level combinations, and seasonal target assemblages and should jointly consider entrance-depth screening criteria, detectability of entrance flow, the internal mainstream route, and low-velocity resting space rather than evaluate suitability from a single head difference or maximum velocity. Entrance flow must balance attraction and entry, while internal hydraulics should avoid continuous high-velocity barriers and maintain connectivity between passage routes and low-velocity areas [19,22,24]. The present fishway has no dedicated auxiliary attraction flow, and supplementary attraction flow may be considered during periods of weak head difference to strengthen the entrance flow cue [18]. During operation, priority passage periods can be identified from tidal forecasts and real-time water levels [64], and entrance and exit gate openings, supplementary discharge, and entrance attraction flow may be adjusted according to the seasonal migration periods of target fishes. When small- and medium-sized fishes predominate, operation should prioritize a continuous water column and lower sustained hydraulic loading. A broader operating range may be appropriate when larger fishes predominate, but its limits should be determined from target-species capacities and field observations. Studies of tidal barriers show that tide-stage-specific operation can increase passage opportunities, whereas mismatch between migration activity and favorable hydraulics can cause holding and delay [27,65].

Several limitations should be considered when interpreting these results. Hydraulic validation covered surface velocities at 16 points in one pool at a seaward tidal level of 4.14 m, and the mesh comparison focused on longitudinal water levels. Further validation is needed for near-bed velocity peaks and entrance wetting and drying. The five simulations characterize hydraulic conditions at selected fixed tidal levels, with transient flow processes remaining to be examined. Sonar passage counts represent crossing events at the exit monitoring section; individual entry times, residence periods, repeat crossings after tracking was lost, and passage success through the entire fishway remain unresolved. Fish–debris classification also requires independent validation. Body lengths were measured from clearly resolved targets on four dates, including two June dates representing March–June, so their interpretation is limited to the sampled targets and dates. Sampling was unbalanced across monitoring periods and years, and daily mean air temperature provided an approximate environmental covariate for unmeasured water temperature. Residual temporal dependence may remain among consecutive observations after accounting for variation among dates, while high-count dates, particularly 20 June 2025, may influence descriptive summaries. These considerations support interpreting the findings primarily as temporal patterns of recorded passage and their associations with concurrent tidal and hydraulic conditions. Entry conditions and the biological processes underlying these patterns warrant further investigation.

Monitoring should integrate hydraulic, environmental, and biological processes. Hydraulic measurements should continuously record freshwater and seaward water levels, head difference, the rate of tidal-level change, entrance depth, and velocities in critical zones. Environmental monitoring should include temperature, salinity, turbidity, dissolved oxygen, chlorophyll *a*, phytoplankton, and zooplankton; several of these variables have been used to explain estuarine fish passage, habitat use, and abundance [40,66]. Biological monitoring should identify species, body size, abundance, movement direction, and passage stage whenever possible. A second monitoring section should be established at the seaward entrance and synchronized with the existing section near the freshwater exit. Combined with individual marking and tracking, these observations would allow entry and completion records to be linked, transit times to be measured, and repeated crossings by the same fish to be identified. Additional observations at critical internal sections would help locate delays and resting areas. In the present study, continuous trajectory review linked successive detections while targets remained visible, and the reported passage intensities describe crossing events at the monitoring section. Future monitoring would extend the analysis of completed passage to separate assessments of attraction, entry, and passage completion, with hydraulic and behavioral observations used to identify bottlenecks [10,11,12,13,14,15].

## 5. Conclusions

Long-term tidal records, fishway sonar monitoring, and three-dimensional hydrodynamic simulation were combined to characterize tidal responses of fishway hydraulics and seasonal passage through the Cao’e River fishway. The principal conclusions are as follows:

(1) The hydraulic effect of seaward tidal level was spatially nonuniform, and the entrance was the principal hydraulically sensitive zone at low tide. As the seaward level decreased, the water-surface slope increased and entrance depth declined. The simulations indicated local loss of entrance connectivity at a seaward level of 0.20 m. At a seaward level of 1.16 m, mean entrance depth was approximately 0.55 m. On the monitoring dates, the seaward-level reference corresponding to the adopted entrance-depth screening criterion was met or exceeded for 5.7–6.2 h per 12 h cycle. At a seaward level of 1.16 m, velocities below 0.3 m/s occupied more than 80% of the seaward resting-pool area at the notch elevation. At the orifice elevation, mainstream velocities reached 0.7 to 1.0 m/s, with local values above 1.0 m/s.
(2) Fish passage varied seasonally in both peak tidal stage and diel rhythm. Upstream crossings near the freshwater-side exit were concentrated around high tide during March–June and around low tide during September–November. Total passage intensities during the late flood and early ebb stages in March–June were 371 and 344 ind/h, respectively, whereas the early flood and late ebb stages in September–November reached 57 and 43 ind/h. Passage in both periods was concentrated during daylight, with hourly maxima of 579 and 117 ind/h, respectively. Lower passage intensity during September–November remained statistically supported in both directions after adjustment for monitoring effort, the other model covariates, and variation among monitoring dates.
(3) Upstream crossing intensity recorded near the freshwater-side exit was associated with distinct combinations of head difference and tidal-level change rate, and differences between tidal categories varied among tidal stages. During March–June, the principal high-value region occurred during the ebb tide at *dZ*/*dt* = −1.5 to −1.1 m/h and Δ*H* = 2.25–3.75 m, with a regional mean upstream intensity of approximately 548 ind/h and a maximum of 1252 ind/h. During September–November, elevated intensities occurred under small positive to reverse heads during flood tide and large positive heads during ebb tide, with regional means of approximately 54 and 41 ind/h, respectively. These distributions describe hydraulic conditions concurrent with upstream crossings near the freshwater-side exit. Passage was generally more active under spring tides, with differences varying among tidal stages.
(4) Effective passage through an estuarine fishway depends on dynamic matching between tidal-hydraulic conditions and seasonal migration characteristics. Mean sonar-estimated target length was 17.6 ± 3.4 cm during March–June and 26.8 ± 6.7 cm during September–November. These size differences, together with historical assemblage information, suggest that seasonal passage patterns may reflect differences in the use of available hydraulic conditions. Design and operation should maintain entrance connectivity and connections between internal passage routes and resting space, align operating periods with seasonal passage activity and tidal conditions, and integrate hydraulic, environmental, and biological monitoring.

## Figures and Tables

**Figure 1:**
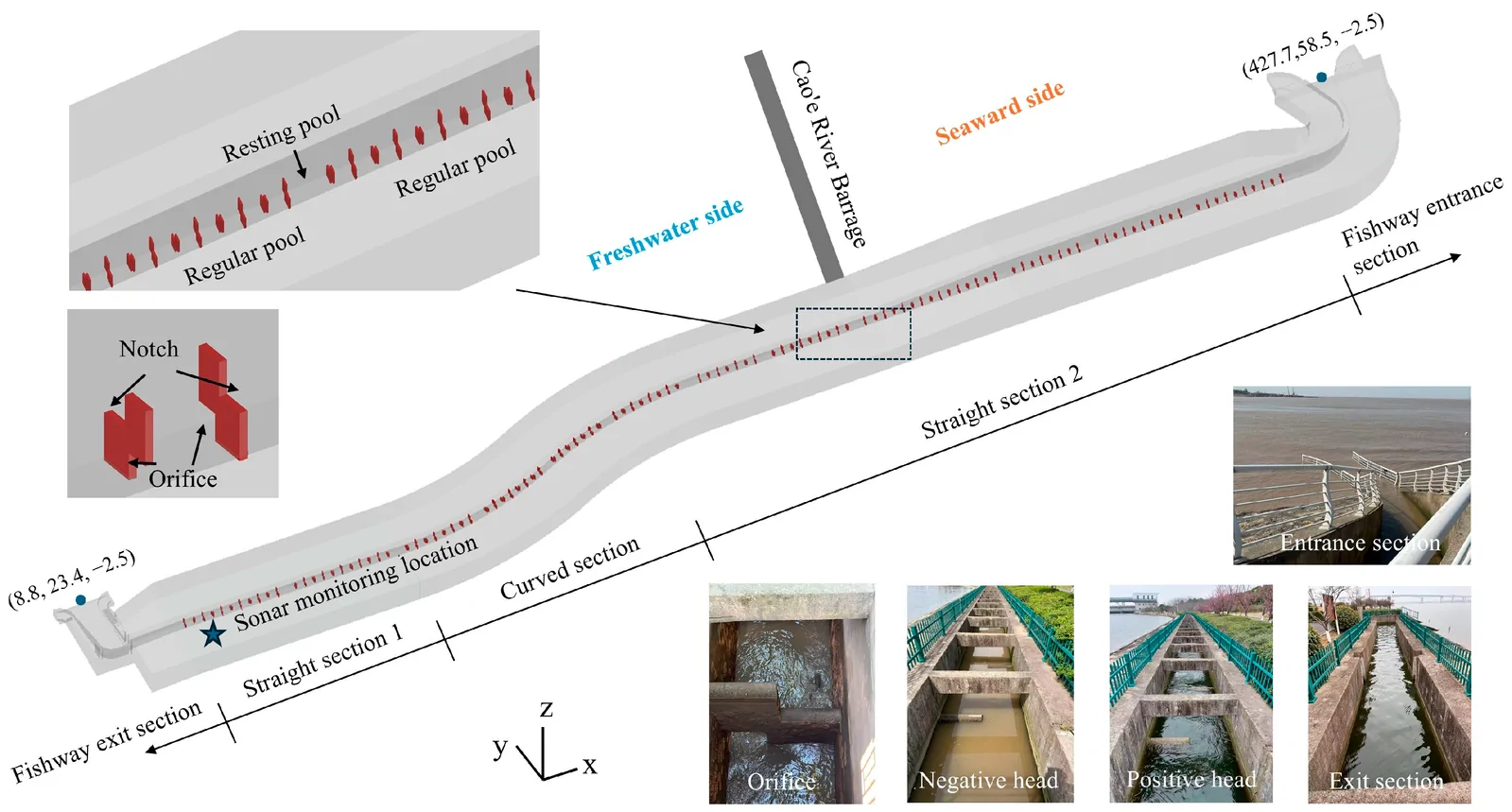
Overall layout, typical structures, and sonar-monitoring location of the right-bank fishway at the Cao’e River Sluice.

**Figure 2:**
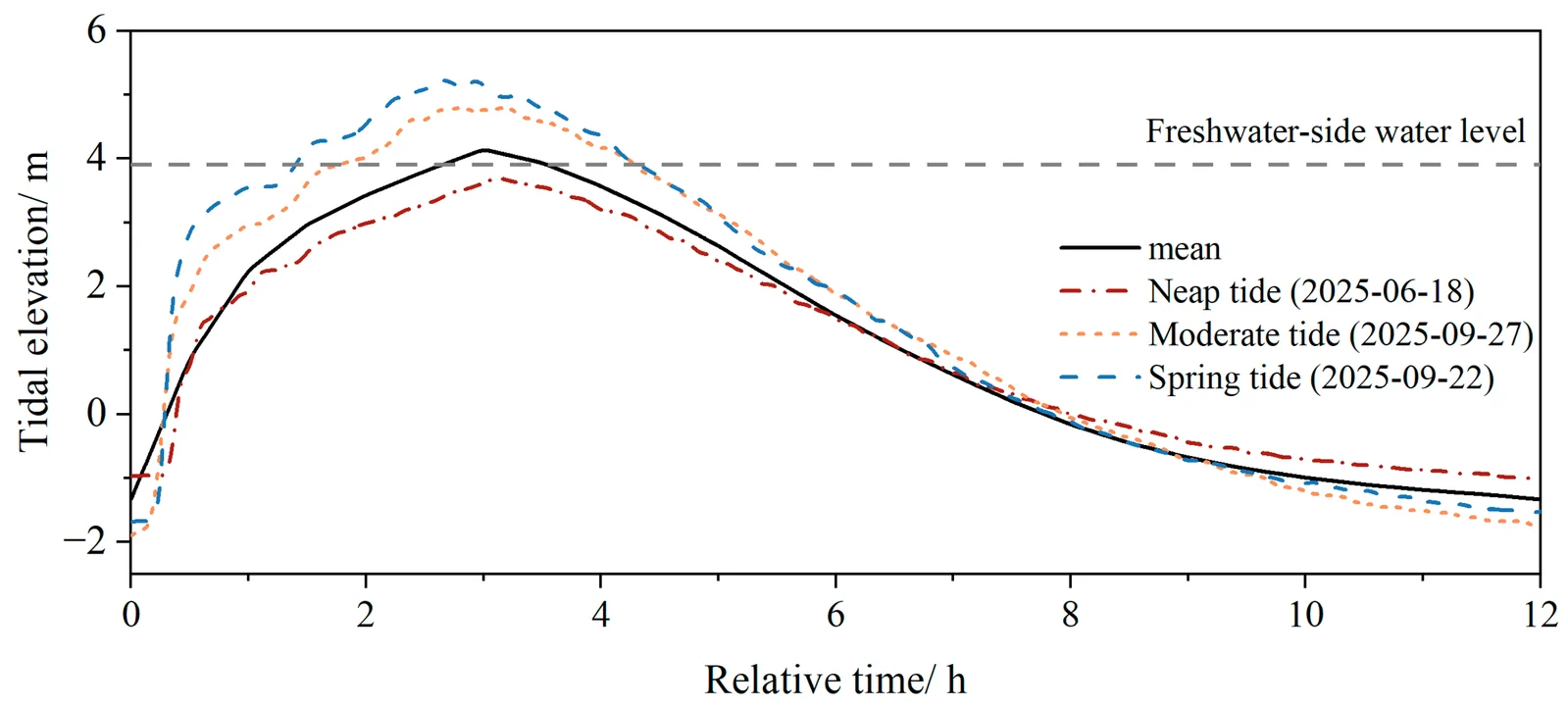
Mean seaward tidal cycle at the Cao’e River Sluice and representative tidal cycles under different tidal categories.

**Figure 3:**
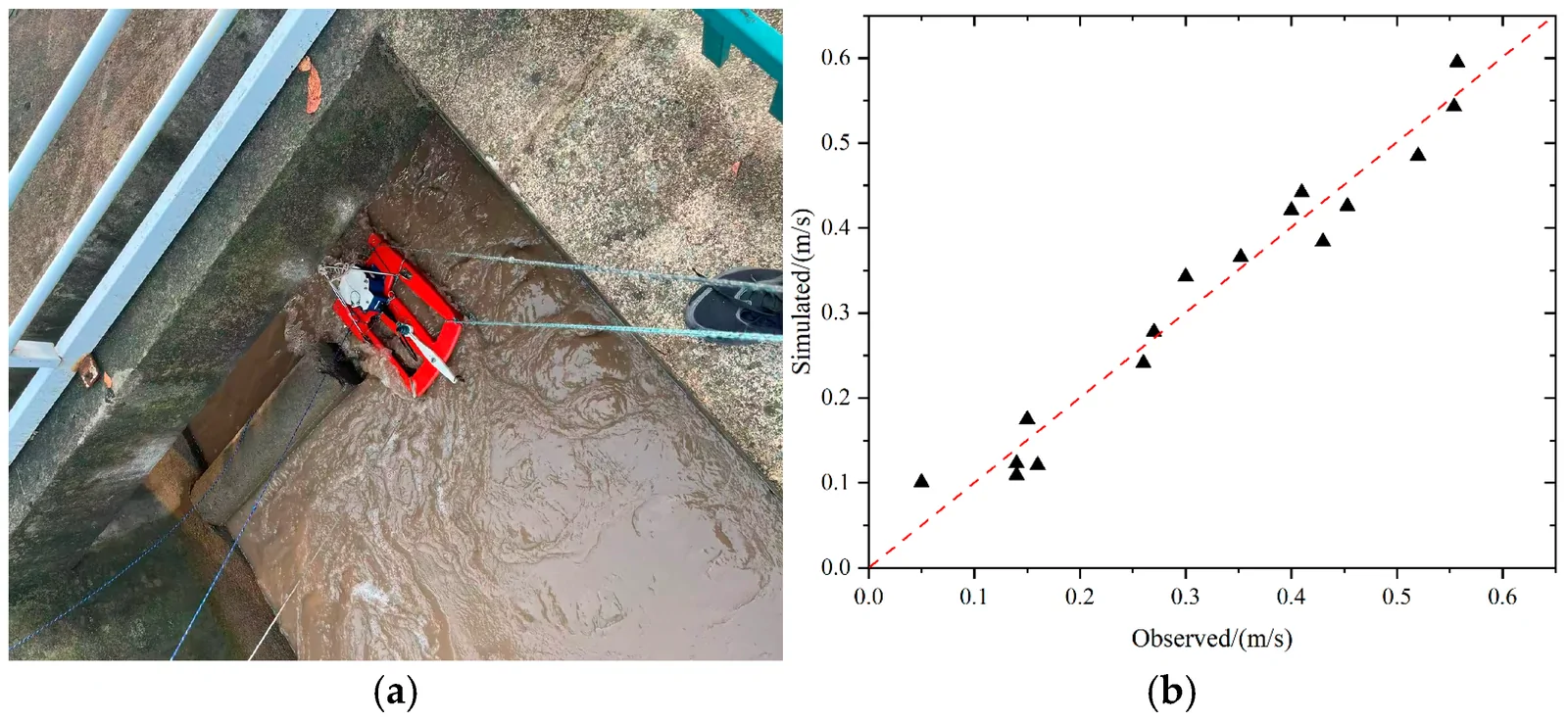
Velocity validation for the numerical model. (**a**) Field velocity measurements; (**b**) comparison between measured and simulated velocities at 16 measurement points. Black triangles represent paired velocity values, and the red dashed line indicates the 1:1 line.

**Figure 4:**
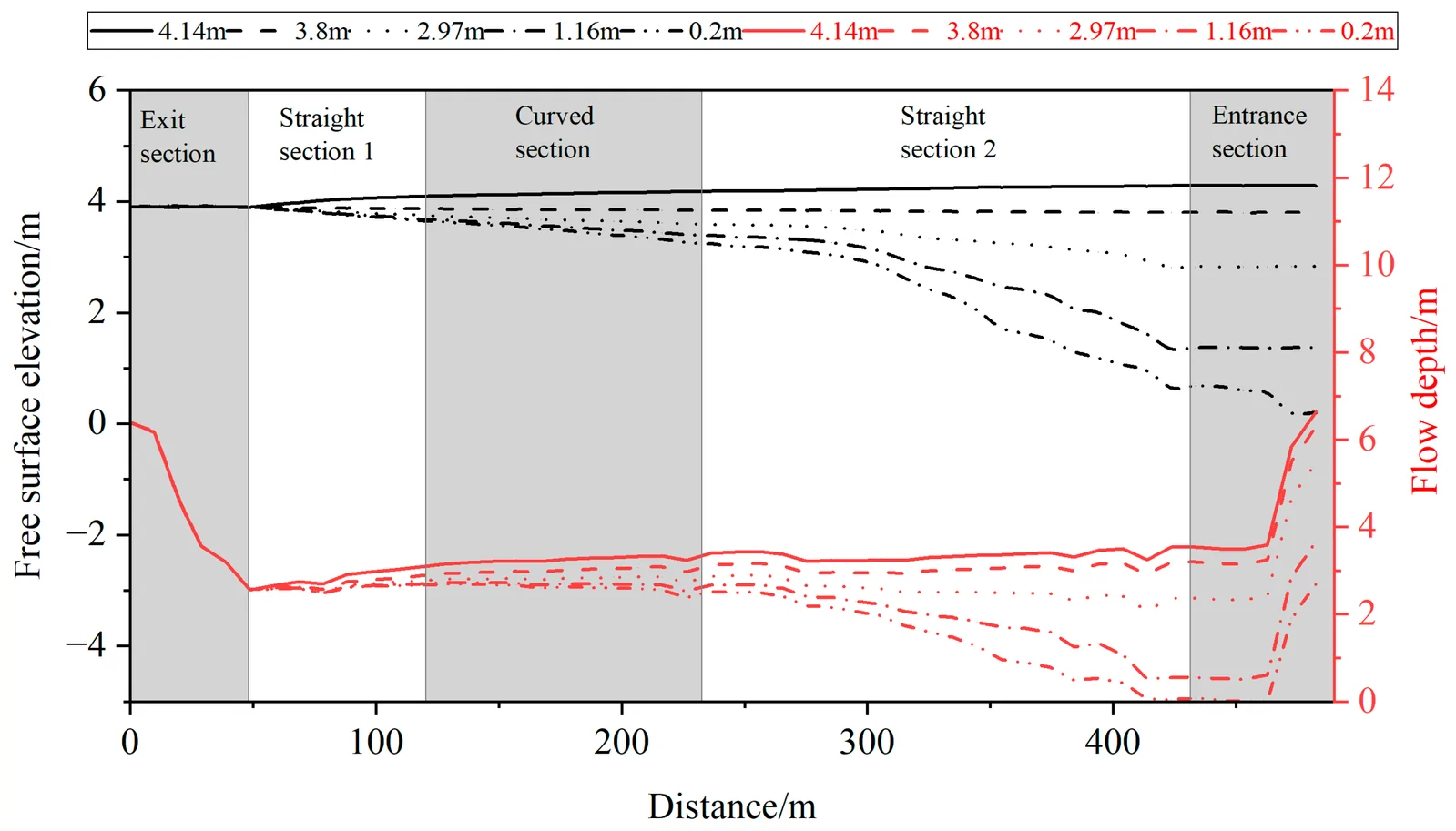
Longitudinal water-surface profiles and water depths under different seaward tidal levels.

**Figure 5:**
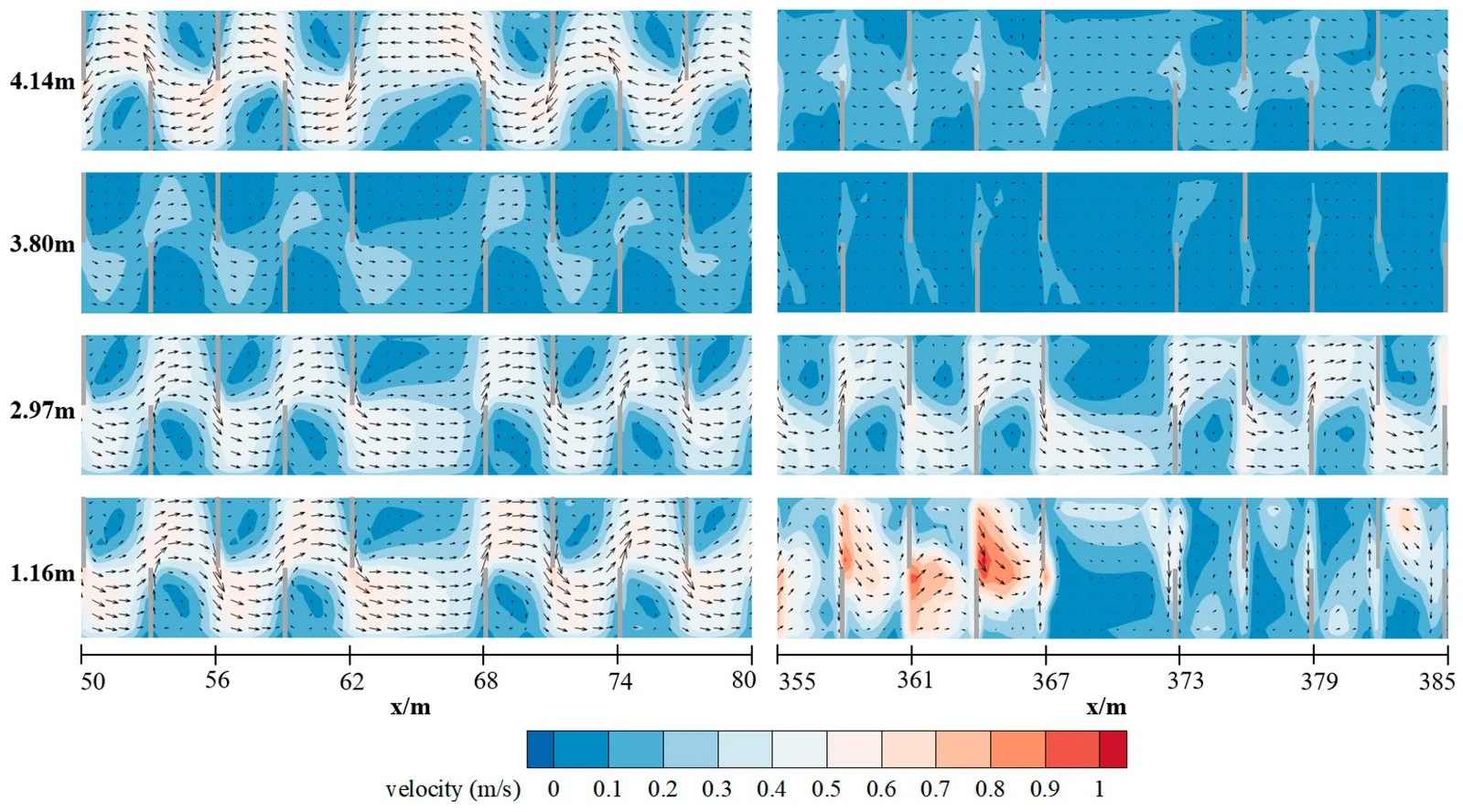
Velocity distributions at the elevation of the notches in representative sections under different seaward tidal levels. The left column shows the freshwater-side section (*z* = 3.1 m), and the right column shows the seaward section (*z* = 2.1 m). Arrows indicate flow direction, and their lengths represent relative velocity magnitude.

**Figure 6:**
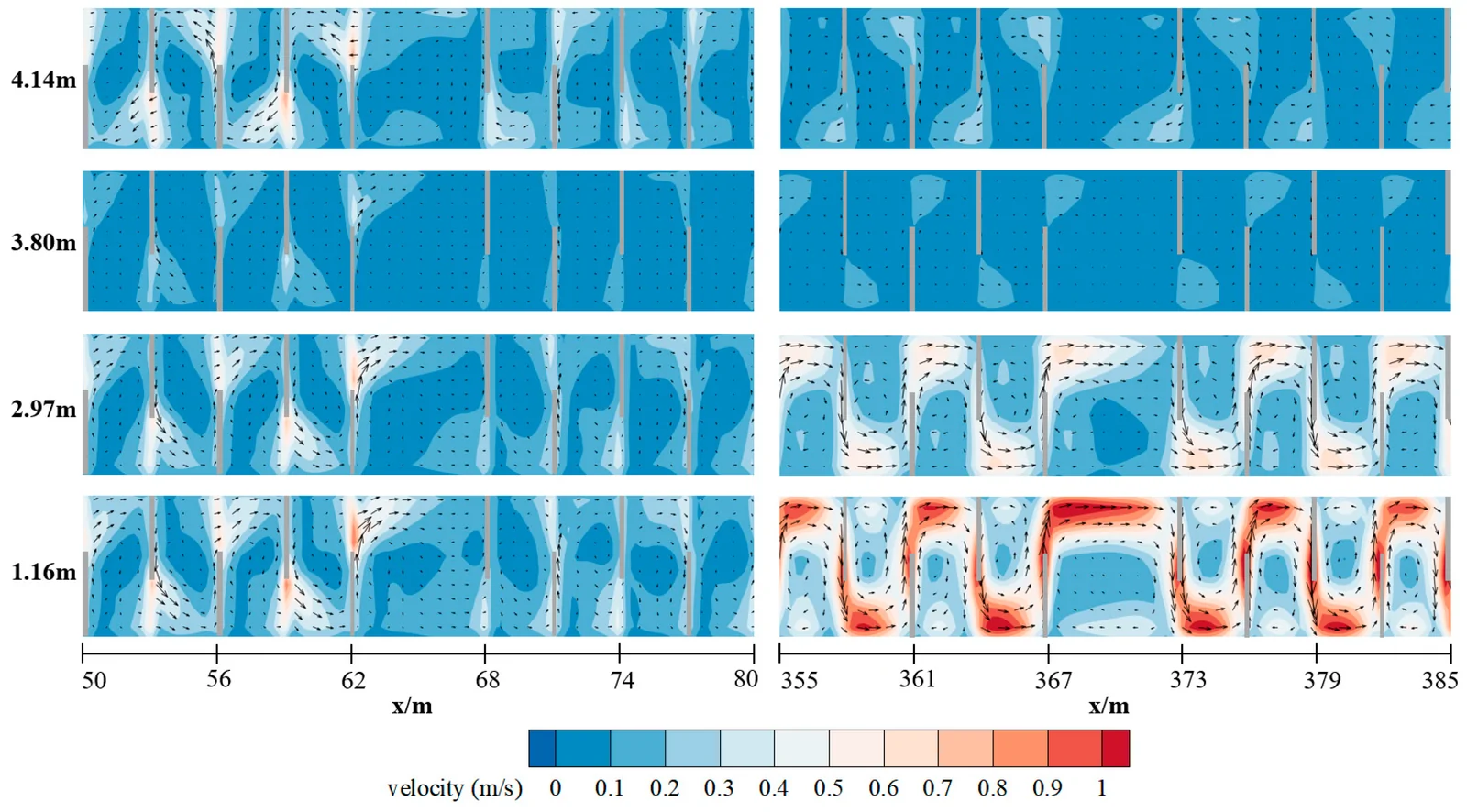
Velocity distributions at the elevation of the orifices in representative sections under different seaward tidal levels. The left column shows the freshwater-side section (*z* = 2.1 m), and the right column shows the seaward section (*z* = 1.1 m). Arrows indicate flow direction, and their lengths represent relative velocity magnitude.

**Figure 7:**
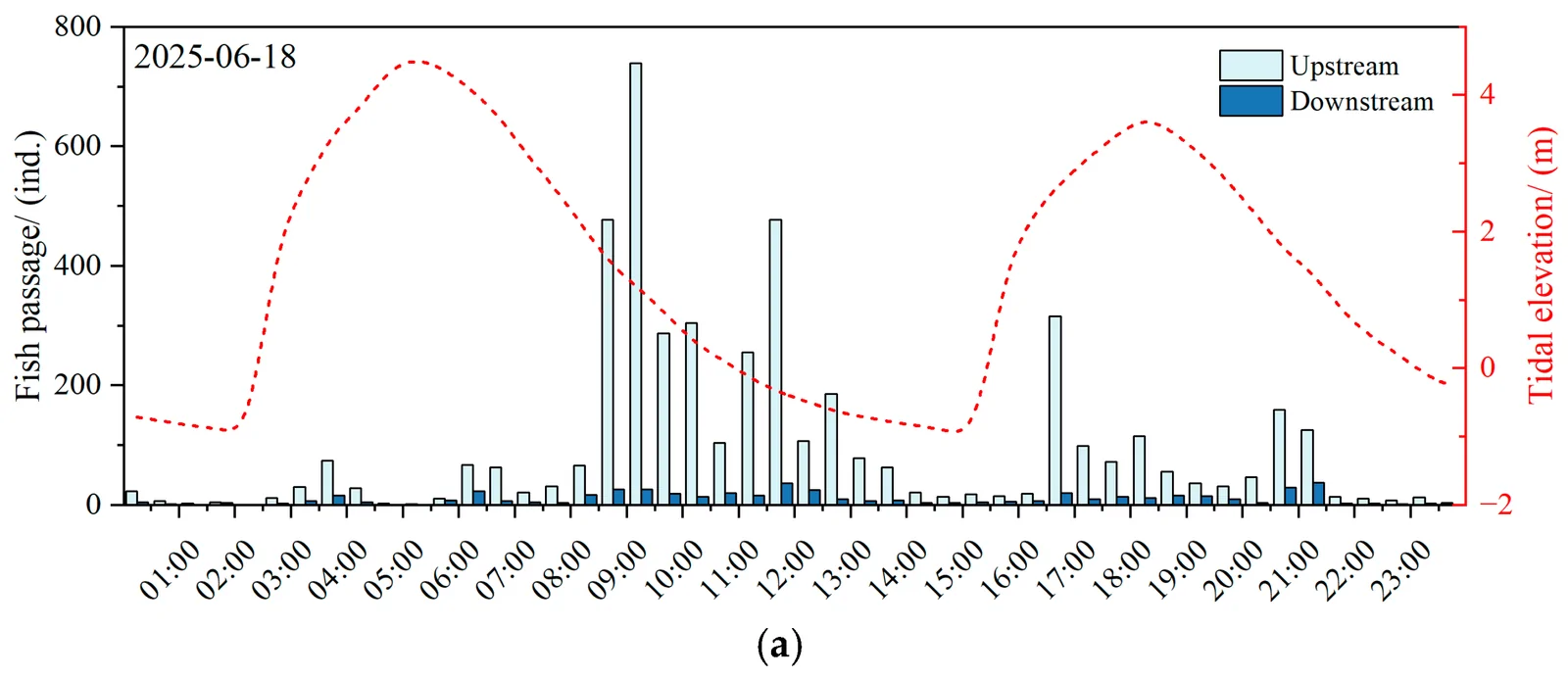

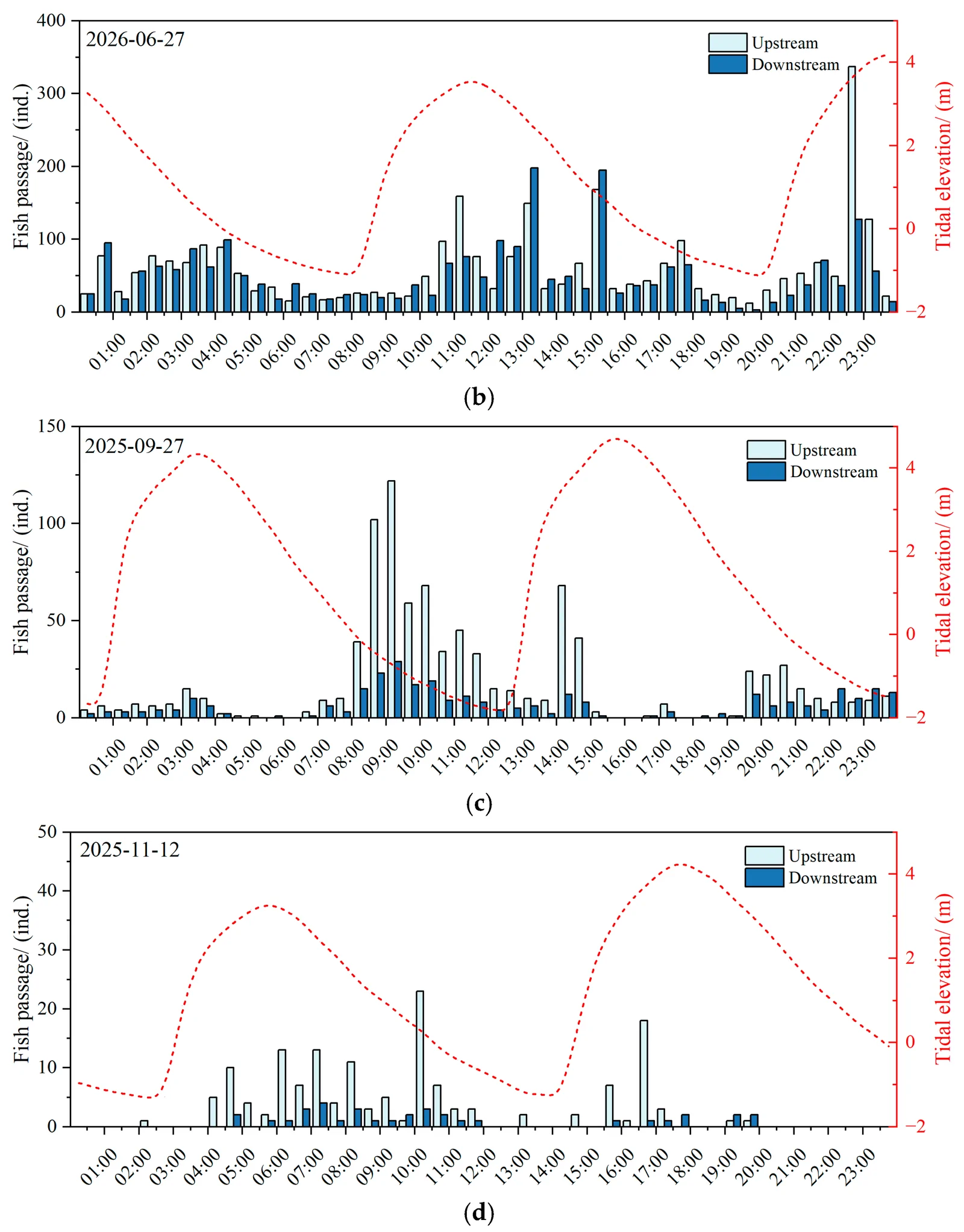
Diel variation in upstream and downstream passage abundance and seaward tidal level on representative monitoring dates: (**a**) 18 June 2025; (**b**) 27 June 2026; (**c**) 27 September 2025; (**d**) 12 November 2025. The red dashed line indicates the seaward tidal level (right axis).

**Figure 8:**
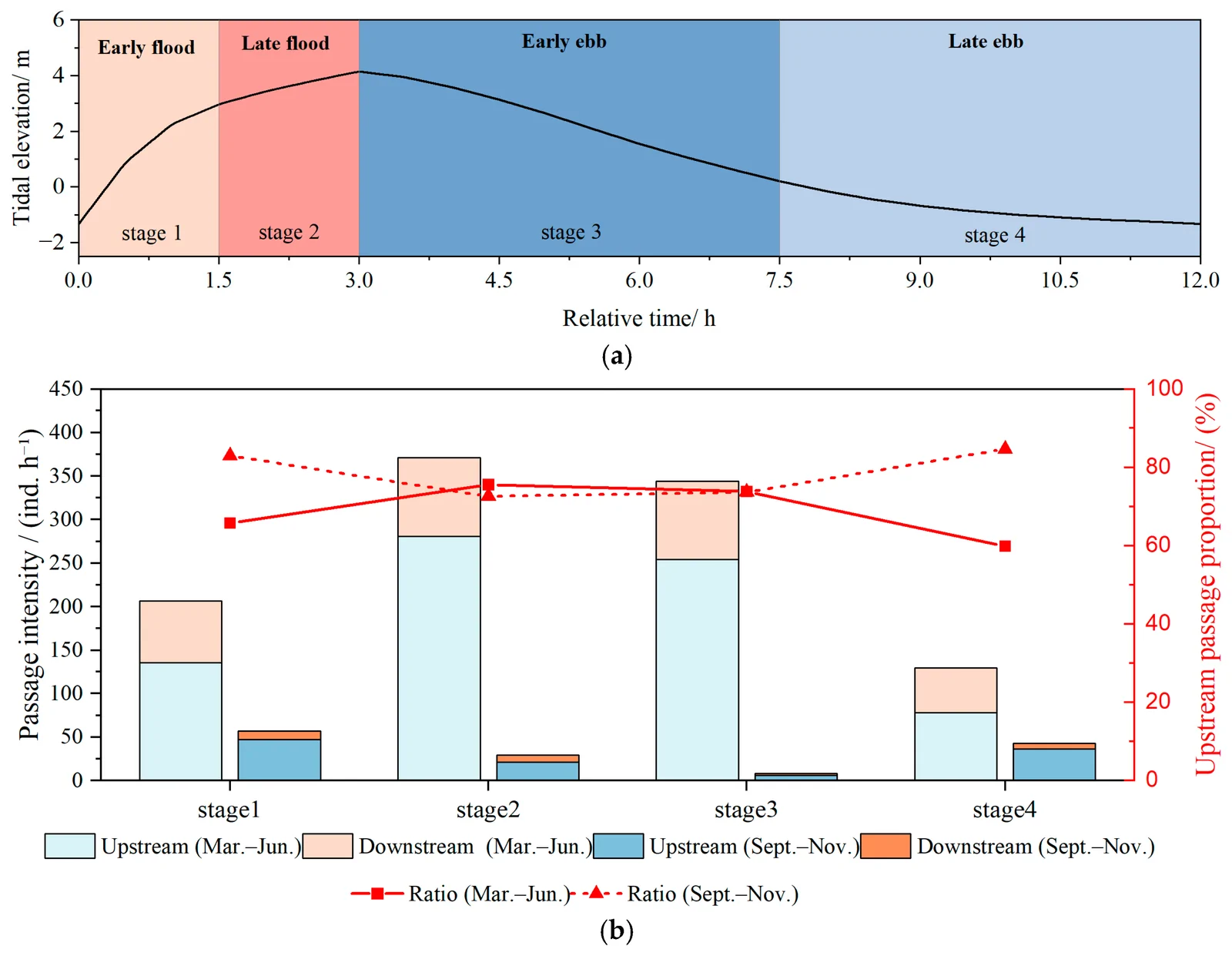
Tidal-stage definition and stage distributions of passage intensity during the two seasonal periods. (**a**) Division of the tidal cycle into four stages; (**b**) upstream and downstream passage intensities and upstream proportions by tidal stage during March–June and September–November.

**Figure 9:**
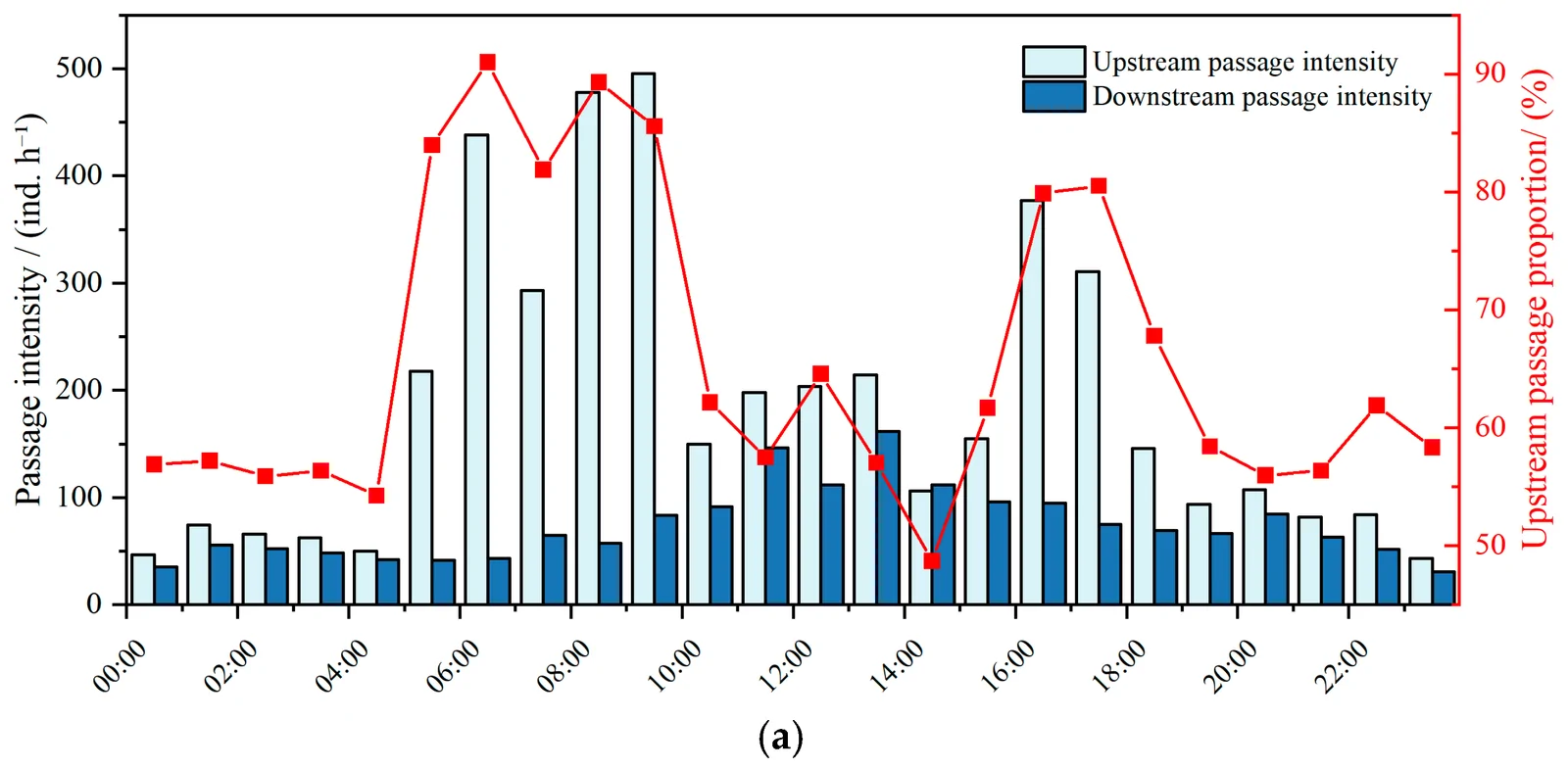

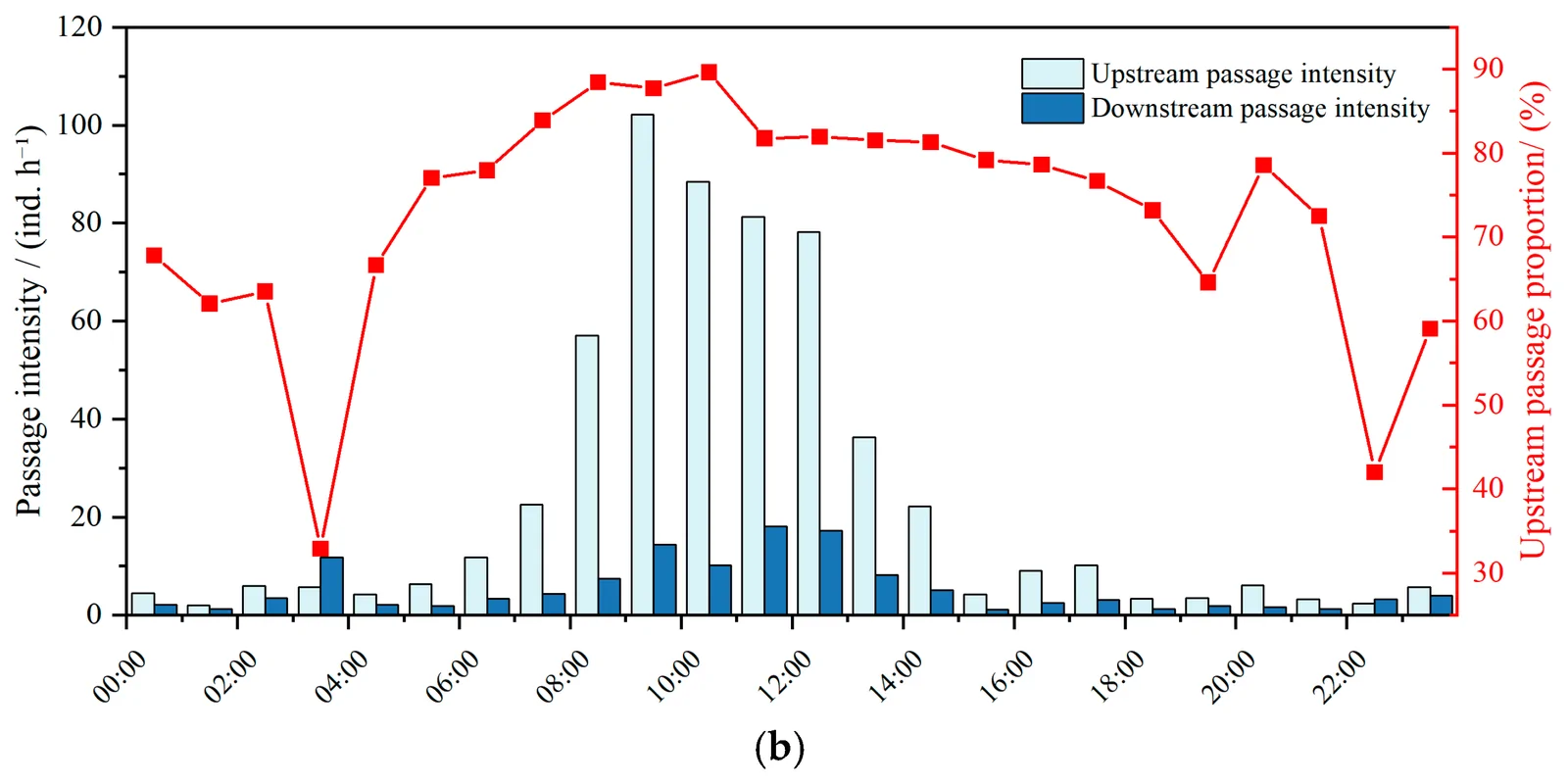
Diel variation in upstream and downstream passage intensities and upstream proportion during the two seasonal periods: (**a**) March–June; (**b**) September–November. The red squares and connecting line indicate the upstream passage proportion (right axis).

**Figure 10:**
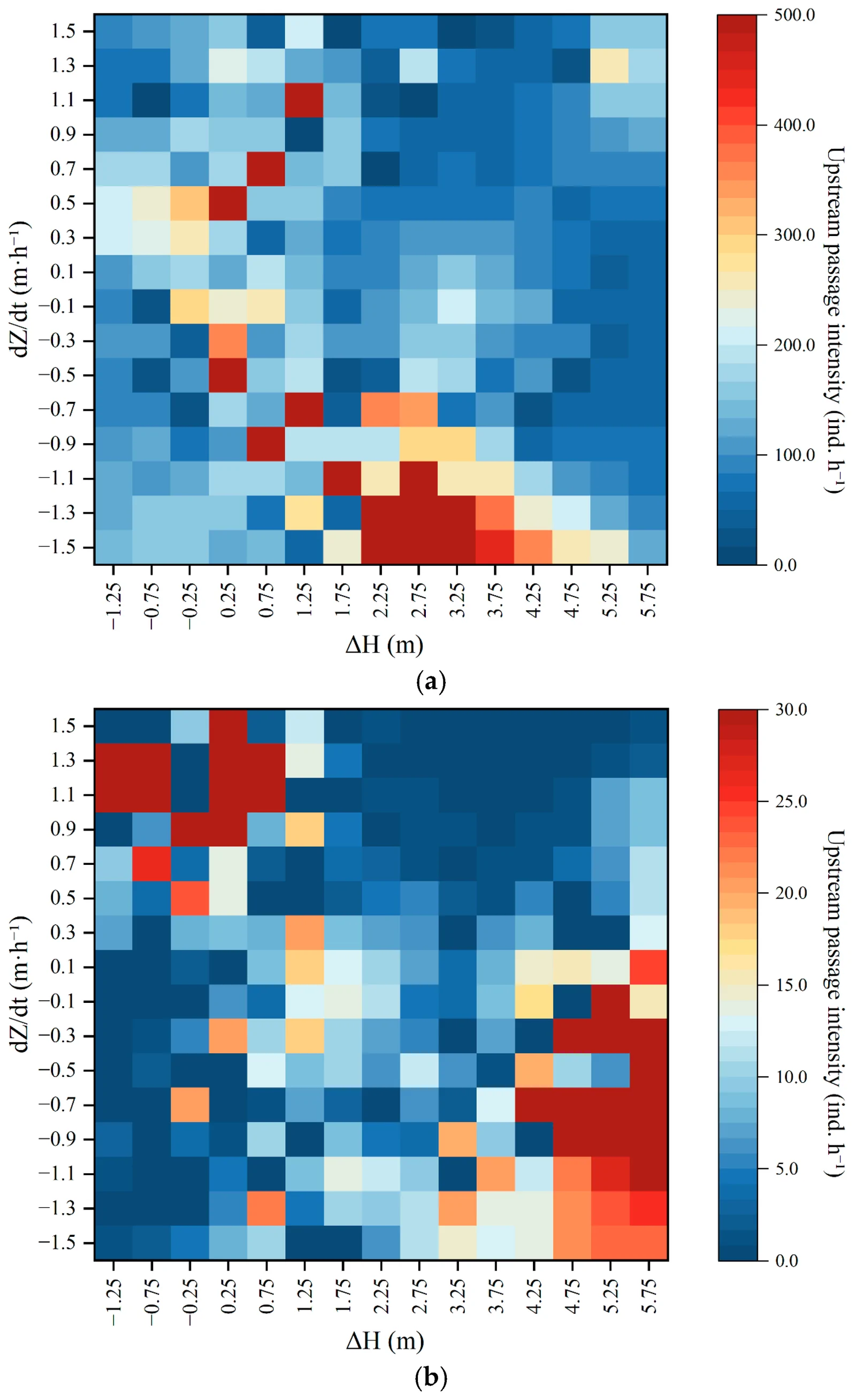
Two-dimensional response of upstream passage intensity to head difference and the rate of tidal-level change. (**a**) March–June; (**b**) September–November. Hydraulic variables correspond to the times at which upstream targets crossed the exit monitoring section.

**Table 1:** Historical composition and size characteristics of major fish taxa sampled in the right-bank fishway of the Cao’e River Sluice in 2017.

Species	May–June (*n*)	September–November (*n*)	Body Length Range (cm)	Body Weight Range (g)
Japanese grenadier anchovy (*Coilia ectenes*)	795	16	10–27	8–90
Sharpbelly (*Hemiculter leucisculus*)	681	0	8–19	7–90
Small yellow croaker (*Larimichthys polyactis*)	44	0	3.5–7	2–30
Spotted seabass (*Lateolabrax maculatus*)	16	15	2.5–40	1–1000
Crucian carp (*Carassius auratus*)	31	27	9–25	25–300
Silver carp (*Hypophthalmichthys molitrix*)	0	30	15–55	50–2000
Bighead carp (*Aristichthys nobilis*)	0	38	15–60	52–2100
Topmouth culter (*Culter alburnus*)	0	16	13–38	12–650
Mongolian culter (*Culter mongolicus*)	1	15	14–30	50–150
Redlip mullet (*Liza haematocheila*)	0	8	15–30	30–500

**Table 2:** Numerical-simulation scenarios under different seaward tidal levels.

Case	Seaward Tidal Level (*H_s_*)/m	Head Difference (Δ*H*)/m	Representative Head Condition	Description
A1	4.14	−0.24	Reverse head	Mean high-tide level
A2	3.80	0.10	Small positive head	Near high tide
A3	2.97	0.93	Moderate positive head	Approximately 1.5 h into flood tide and 4.7 h into ebb tide on the mean tidal curve
A4	1.16	2.74	Large positive head	Mean tidal level during the study period
A5	0.20	3.70	Very large positive head	Effective loss of hydraulic connectivity at the entrance

**Table 3:** Descriptive comparison of upstream passage intensity between spring tides and moderate–neap tides during the four tidal stages.

Monitoring Period	Tidal Stage	Spring Tides			Moderate–Neap Tides			*R*_S/MN_
		*n*_c_	*T*_obs_	*I*_U_	*n*_c_	*T*_obs_	*I*_U_	
March–June	Early flood	10	13.5	162	15	21.5	120	1.35
	Late flood	10	12.5	239	15	23.5	303	0.79
	Early ebb	9	38.5	276	15	62.5	241	1.14
	Late ebb	9	32	101	15	60.5	65	1.55
September–November	Early flood	5	6.5	158	12	19	10	16.45
	Late flood	6	8.5	30	12	20	18	1.69
	Early ebb	6	27.5	6	12	55.5	6	1.05
	Late ebb	6	29.5	70	13	49.5	16	4.39

Note: *n*_c_, number of tidal cycles contributing at least one valid observation to the corresponding cell; *T*_obs_, actual monitoring duration (h); *I*_U_, upstream passage intensity (ind/h), calculated by dividing the cumulative number of recorded upstream crossing events by the actual monitoring duration; S, spring tides; MN, moderate–neap tides; *R*_S/MN_, descriptive ratio of upstream passage intensity under spring tides to that under moderate–neap tides. Counts include partially monitored tidal cycles and represent the distinct cycles contributing observations to each tidal stage. A cycle may contribute to multiple stages, so these counts should not be summed across rows.

**Table 4:** Results of the negative binomial generalized linear mixed models for upstream and downstream passage intensity.

Factor	Contrast or Increment	Upstream Passage		Downstream Passage	
		*R*_adj,U_ (95% CI)	*p*	*R*_adj,D_ (95% CI)	*p*
Monitoring period	September–November vs. March–June at early flood	0.177 (0.089–0.355)	<0.001	0.102 (0.043–0.245)	<0.001
	September–November vs. March–June at late flood	0.089 (0.045–0.176)	<0.001	0.073 (0.031–0.175)	<0.001
	September–November vs. March–June at early ebb	0.033 (0.019–0.059)	<0.001	0.024 (0.011–0.055)	<0.001
	September–November vs. March–June at late ebb	0.114 (0.064–0.204)	<0.001	0.074 (0.033–0.166)	<0.001
Tidal stage, March–June	Late flood vs. early flood	1.669 (1.067–2.611)	0.025	1.499 (1.036–2.169)	0.032
	Early ebb vs. early flood	1.141 (0.731–1.782)	0.562	1.114 (0.767–1.620)	0.571
	Late ebb vs. early flood	0.453 (0.270–0.759)	0.003	0.578 (0.382–0.876)	0.010
Tidal stage, September–November	Late flood vs. early flood	0.839 (0.480–1.466)	0.537	1.078 (0.643–1.807)	0.776
	Early ebb vs. early flood	0.214 (0.120–0.382)	<0.001	0.264 (0.156–0.446)	<0.001
	Late ebb vs. early flood	0.291 (0.153–0.555)	<0.001	0.417 (0.236–0.736)	0.003
Tidal category	Spring vs. moderate–neap tides	1.702 (1.010–2.866)	0.046	1.879 (0.868–4.070)	0.110
Diel period	06:00–12:00 vs. 00:00–06:00	5.519 (4.187–7.275)	<0.001	2.702 (2.139–3.413)	<0.001
	12:00–18:00 vs. 00:00–06:00	3.035 (2.321–3.968)	<0.001	2.622 (2.069–3.322)	<0.001
	18:00–24:00 vs. 00:00–06:00	1.415 (1.076–1.859)	0.013	1.343 (1.056–1.708)	0.016
Δ*H*	Increase of 1 m	1.074 (0.978–1.179)	0.137	1.032 (0.954–1.117)	0.429
*dZ*/*dt*	Increase of 1 m/h	0.930 (0.847–1.021)	0.128	0.939 (0.867–1.018)	0.129
Temperature	Increase of 1 °C	1.135 (1.073–1.202)	<0.001	1.057 (0.971–1.150)	0.198

Notes: *R*_adj,U_ and *R*_adj,D_ denote the adjusted passage-intensity ratios for upstream and downstream passage, respectively; CI, confidence interval; Δ*H*, head difference; and *dZ*/*dt*, tidal-level change rate. Temperature refers to the daily mean air temperature used as an approximate environmental covariate. For categorical factors, each ratio compares the first condition listed with the second condition; for continuous variables, each ratio corresponds to the stated increment. Ratios and confidence limits for the continuous variables were converted from standardized model coefficients using standard deviations of 1.984 m for Δ*H*, 1.469 m/h for *dZ*/*dt*, and 4.743 °C for daily mean air temperature. A ratio greater than 1 indicates higher passage intensity, and a ratio below 1 indicates lower passage intensity. Because monitoring period and tidal stage were included as an interaction, their contrasts are presented separately for each tidal stage and monitoring period. The logarithm of actual monitoring duration was included as an offset, and monitoring date was specified as a random intercept. *p* values were obtained using two-sided Wald tests.

## Data Availability

The fish passage monitoring and tidal-level data are provided in Dataset S1. Additional data supporting the findings of this study are available from the corresponding authors upon reasonable request.

## Supplementary Materials

The following supporting information can be downloaded at: https://www.mdpi.com/article/10.3390/ani16193014/s1, Figure S1: representative sonar images; Figure S2: mesh comparison of water-surface profiles; Figure S3: ADCP measurement-point layout; Figure S4: notch-level velocity distributions with contour lines; Figure S5: orifice-level velocity distributions with contour lines; Figure S6: length-frequency distributions of sonar targets; Table S1: historical fishway catch data; Table S2: hydraulic characteristics of the simulation scenarios; Table S3: entrance-depth screening durations; Table S4: correlations among model variables; Table S5: sensitivity analysis at the 1 h resolution; Table S6: tidal-stage distributions under assumed transit times; Table S7: spatial similarity under assumed transit times; Dataset S1: fish passage and tidal-level data.

## References

[1] Livernois M.C., Ogburn M.B., Legett H.D., Richie K.D., Aguilar R., Heggie K., Furey N.B. (2025). Tracking animal movements via collaborative acoustic telemetry networks: Multiscale habitat use, phenology, and management insights. J. Fish Biol..

[2] Beck M.W., Heck K.L., Able K.W., Childers D.L., Eggleston D.B., Gillanders B.M., Halpern B., Hays C.G., Hoshino K., Minello T.J. (2001). The identification, conservation, and management of estuarine and marine nurseries for fish and invertebrates. Bioscience.

[3] Gillanders B.M., Able K.W., Brown J.A., Eggleston D.B., Sheridan P.F. (2003). Evidence of connectivity between juvenile and adult habitats for mobile marine fauna: An important component of nurseries. Mar. Ecol. Prog. Ser..

[4] Able K.W. (2005). A re-examination of fish estuarine dependence: Evidence for connectivity between estuarine and ocean habitats. Estuar. Coast. Shelf Sci..

[5] Grill G., Lehner B., Thieme M., Geenen B., Tickner D., Antonelli F., Babu S., Borrelli P., Cheng L., Crochetiere H. (2019). Mapping the world’s free-flowing rivers. Nature.

[6] Belletti B., Garcia de Leaniz C., Jones J., Bizzi S., Börger L., Segura G., Castelletti A., van de Bund W., Aarestrup K., Barry J. (2020). More than one million barriers fragment Europe’s rivers. Nature.

[7] Franklin P.A., Bašić T., Davison P.I., Dunkley K., Ellis J., Gangal M., González-Ferreras A.M., Gutmann Roberts C., Hunt G., Joyce D. (2024). Aquatic connectivity: Challenges and solutions in a changing climate. J. Fish Biol..

[8] Tickner D., Opperman J.J., Abell R., Acreman M., Arthington A.H., Bunn S.E., Cooke S.J., Dalton J., Darwall W., Edwards G. (2020). Bending the curve of global freshwater biodiversity loss: An emergency recovery plan. Bioscience.

[9] Sun J., Galib S.M., Lucas M.C. (2021). Rapid response of fish and aquatic habitat to removal of a tidal barrier. Aquat. Conserv. Mar. Freshw. Ecosyst..

[10] Roscoe D.W., Hinch S.G. (2010). Effectiveness monitoring of fish passage facilities: Historical trends, geographic patterns and future directions. Fish Fish..

[11] Bunt C.M., Castro-Santos T., Haro A. (2012). Performance of fish passage structures at upstream barriers to migration. River Res. Appl..

[12] Noonan M.J., Grant J.W.A., Jackson C.D. (2012). A quantitative assessment of fish passage efficiency. Fish Fish..

[13] Silva A.T., Lucas M.C., Castro-Santos T., Katopodis C., Baumgartner L.J., Thiem J.D., Aarestrup K., Pompeu P.S., O’Brien G.C., Braun D.C. (2018). The future of fish passage science, engineering, and practice. Fish Fish..

[14] Hershey H. (2021). Updating the consensus on fishway efficiency: A meta-analysis. Fish Fish..

[15] Sun J., Tan J., Zhang Q., Shen Y., Shi J., Zhang H., Shi X. (2023). Attraction and passage efficiency for salmonids and non-salmonids based on fishway: A meta-analysis approach. River Res. Appl..

[16] Bice C.M., Huisman J., Kimball M.E., Mallen-Cooper M., Zampatti B.P., Gillanders B.M. (2023). Tidal barriers and fish—Impacts and remediation in the face of increasing demand for freshwater and climate change. Estuar. Coast. Shelf Sci..

[17] Ye Z., Lian X., Bai F., Hao D., Li D., Fang Z. (2023). Response of upstream behavior and hydrodynamic factors of *Anguilla japonica* in a combined bulkhead fishway under tidal conditions. Water.

[18] Schütz C., Henning M., Czerny R., Herbst M., Pitsch M. (2021). Addition of auxiliary discharge into a fishway—A contribution to fishway design at barrages of large rivers. Ecol. Eng..

[19] Schütz C., Czerny R., Herbst M., Henning M., Pitsch M. (2024). Passage through a fishway entrance at different velocities—Results from flume experiments with small non-salmonids. River Res. Appl..

[20] Richer E.E., Fetherman E.R., Krone E.A., Wright F.B., Kondratieff M.C. (2020). Multispecies fish passage evaluation at a rock-ramp fishway in a Colorado transition zone stream. N. Am. J. Fish. Manag..

[21] Lewandoski S.A., Hrodey P., Miehls S., Piszczek P.P., Zielinski D.P. (2021). Behavioral responses of sea lamprey (*Petromyzon marinus*) and white sucker (*Catostomus commersonii*) to turbulent flow during fishway passage attempts. Can. J. Fish. Aquat. Sci..

[22] Wiegleb J., Hirsch P.E., Seidel F., Rauter G., Burkhardt-Holm P. (2023). Impact of hydraulic forces on the passage of round goby (*Neogobius melanostomus*), gudgeon (*Gobio gobio*) and bullhead (*Cottus gobio*) in a vertical slot fish pass. Ecol. Freshw. Fish.

[23] Ke S., Tu Z., Goerig E., Tan J., Cheng B., Li Z., Shi X. (2022). Swimming behaviour of silver carp (*Hypophthalmichthys molitrix*) in response to turbulent flow induced by a D-cylinder. J. Fish Biol..

[24] Zhang D., Xu Y., Deng J., Shi X., Liu Y. (2024). Relationships among the fish passage efficiency, fish swimming behavior, and hydraulic properties in a vertical-slot fishway. Fish. Manag. Ecol..

[25] Alcott D., Goerig E., Rillahan C., He P., Castro-Santos T. (2021). Tide gates form physical and ecological obstacles to river herring (*Alosa* spp.) spawning migrations. Can. J. Fish. Aquat. Sci..

[26] Rillahan C.B., Alcott D., Castro-Santos T., He P. (2021). Activity patterns of anadromous fish below a tide gate: Observations from high-resolution imaging sonar. Mar. Coast. Fish..

[27] Van Wichelen J., Verhelst P., Buysse D., Belpaire C., Vlietinck K., Coeck J. (2021). Glass eel (*Anguilla anguilla* L.) behaviour after artificial intake by adjusted tidal barrage management. Estuar. Coast. Shelf Sci..

[28] Tracey S.R., Hartmann K., McAllister J., Lyle J.M. (2020). Home range, site fidelity and synchronous migrations of three co-occurring, morphologically distinct estuarine fish species. Sci. Total Environ..

[29] Fukushima M., Rand P.S. (2023). Individual variation in spawning migration timing in a salmonid fish—Exploring roles of environmental and social cues. Ecol. Evol..

[30] Burns C.W., Gibeau P., Robichaud D., McCulloch C., Novoa J., Lockert K. (2023). Eulachon migration patterns in the lower Fraser River revealed through acoustic telemetry. Trans. Am. Fish. Soc..

[31] Becker A., Holland M., Smith J.A., Suthers I.M. (2016). Fish movement through an estuary mouth is related to tidal flow. Estuaries Coasts.

[32] Bornman E., Cowley P.D., Adams J.B., Strydom N.A. (2021). Daytime intra-estuary movements and harmful algal bloom avoidance by *Mugil cephalus* (family *Mugilidae*). Estuar. Coast. Shelf Sci..

[33] Giri S., Chanda A., Maity S., Chakraborty K., Hazra S. (2022). Role of tide and lunar phases on the migration pattern of juvenile Hilsa shad (*Tenualosa ilisha*) within a meso-macrotidal estuary. J. Fish Biol..

[34] Koster W., Church B., Crook D., Dawson D., Fanson B., O’Connor J., Stuart I. (2024). Factors influencing migration of short-finned eels (*Anguilla australis*) over 3 years from a wetland system, Lake Condah, south-east Australia, downstream to the sea. J. Fish Biol..

[35] García-Vega A., Ruiz-Legazpi J., Fuentes-Pérez J.F., Bravo-Córdoba F.J., Sanz-Ronda F.J. (2023). Effect of thermo-velocity barriers on fish: Influence of water temperature, flow velocity and body size on the volitional swimming capacity of northern straight-mouth nase (*Pseudochondrostoma duriense*). J. Fish Biol..

[36] Tarena F., Nyqvist D., Katopodis C., Comoglio C. (2025). Computational fluid dynamics in fishway research-a systematic review on upstream fish passage solutions. J. Ecohydraul..

[37] Yan G.J., He X.K., Cao Z.D., Fu S.J. (2013). An interspecific comparison between morphology and swimming performance in cyprinids. J. Evol. Biol..

[38] Yataya K., Izumi M., Azuma N., Marui A. (2020). Swimming ability of Japanese eel (*Anguilla japonica*) elvers. J. Jpn. Soc. Civ. Eng. Ser. G Environ. Res..

[39] Liu Q., Huang L., Luo Y., Liu X., Liu B., Wang R., Bi C., You X. (2025). Flow tolerance of juvenile *Lateolabrax maculatus*. J. Fish. China.

[40] Addicoat R., Tweedley J.R., Ryan T., Cottingham A., Morgan D.L., Lynch K., Beatty S.J. (2025). Determining the fine-scale movement of an estuarine fish through a tidal-exclusion barrier improves the understanding of mass fish mortality risk. Estuar. Coast. Shelf Sci..

[41] Jones P.E., Champneys T., Vevers J., Börger L., Svendsen J.C., Consuegra S., Jones J., Garcia de Leaniz C. (2021). Selective effects of small barriers on river-resident fish. J. Appl. Ecol..

[42] Eggers F., Schiavon A., Calles O., Watz J., Comoglio C., Candiotto A., Nyqvist D. (2025). Fish behaviour in a vertical slot fishway: Multi-species upstream passage success, size selectivity and diel passage patterns in a large Italian river. River Res. Appl..

[43] Wang S., Xiong Y., Zhang H., Song D., Wang Y., Ge H., Zhang C., Liang L., Zhong X. (2024). Recovery of *Coilia nasus* resources after implementation of the 10-year fishing ban in the Yangtze River: Implied from the Yangtze River Estuary and its adjacent sea areas. Front. Mar. Sci..

[44] Gibson R.N. (2003). Go with the flow: Tidal migration in marine animals. Hydrobiologia.

[45] Miró J.M., Megina C., Garel E., Donázar-Aramendía I., Olaya-Ponzone L., García-Gómez J.C. (2022). Mechanisms for longitudinal transport on early life stages in benthic-pelagic fishes within a tide-dominated estuary. Estuar. Coast. Shelf Sci..

[46] Arevalo E., Cabral H.N., Villeneuve B., Possémé C., Lepage M. (2023). Fish larvae dynamics in temperate estuaries: A review on processes, patterns and factors that determine recruitment. Fish Fish..

[47] Zhang H., Wang Y., Liang C., Liu S., Xian W. (2022). Estuarine ichthyoplankton studies—A review. Front. Mar. Sci..

[48] Whitfield A.K., Able K.W., Barletta M., Blaber S.J.M., Harrison T.D. (2023). Life-history guilds of fishes associated with estuaries: Opportunism versus dependency. Estuar. Coast. Shelf Sci..

[49] Takai K., Kuroki M., Shirai K., Kume M., Murakami H., Terashima Y., Mitamura H., Yamashita Y. (2024). River-sea migration patterns of juvenile Japanese seabass influence migrations in subsequent stages. Estuar. Coast. Shelf Sci..

[50] Medo A., Takai K., Kuroki M., Mitamura H., Kume M., Suzuki K.W., Kojima D., Wada T., Yamashita Y. (2025). Riverward migration associated with food acquisition in adult Japanese seabass *Lateolabrax japonicus*: Evidence from stable sulphur isotope ratios and otolith Sr:Ca ratios. J. Fish Biol..

[51] Edwards J.E., Buijse A.D., Winter H.V., Bijleveld A.I. (2025). Seasonal coastal residency and large-scale migration of two grey mullet species in temperate European waters. Mov. Ecol..

[52] Banet N.V., Fieberg J., Sorensen P.W. (2022). Migration, homing and spatial ecology of common carp in interconnected lakes. Ecol. Freshw. Fish.

[53] Catalano A., Coulter A.A., Glover D.C., Garvey J.E. (2025). Evaluating the use of an established invasive species to predict the movement of an encroaching invader. Anim. Biotelem..

[54] Meijer zu Schlochtern M.P., Nagelkerke L.A.J., Buijse A.D., Vriese F.T., Breukelaar A.W., Hop J., Winter H.V. (2026). Upstream passage windows for anadromous fish at a large barrier in the Rhine-Meuse estuary, 1996–2018. Mov. Ecol..

[55] Rourke M.L., Robinson W., Baumgartner L.J., Doyle J., Mallen-Cooper M., Thiem J.D., Broadhurst M.K. (2025). Multiple fishways mostly maintain upstream teleost movement in a south-eastern Australian river. Front. Fish Sci..

[56] Castro-Santos T., Haro A. (2003). Quantifying migratory delay: A new application of survival analysis methods. Can. J. Fish. Aquat. Sci..

[57] Castro-Santos T. (2004). Quantifying the combined effects of attempt rate and swimming capacity on passage through velocity barriers. Can. J. Fish. Aquat. Sci..

[58] Castro-Santos T. (2005). Optimal swim speeds for traversing velocity barriers: An analysis of volitional high-speed swimming behavior of migratory fishes. J. Exp. Biol..

[59] Haro A., Castro-Santos T., Noreika J., Odeh M. (2004). Swimming performance of upstream migrant fishes in open-channel flow: A new approach to predicting passage through velocity barriers. Can. J. Fish. Aquat. Sci..

[60] Liao J.C. (2007). A review of fish swimming mechanics and behaviour in altered flows. Philos. Trans. R. Soc. B Biol. Sci..

[61] Ruiz-Coello M.X., Sanz-Ronda F.J., Bottacin-Busolin A., Fuentes-Pérez J.F. (2026). Evaluation of the Iberian barbel resting behavior in vertical-slot fishways: A two-dimensional Eulerian–Lagrangian agent-based approach. Adv. Water Resour..

[62] Crawford R.M.B., Gee E., Hicks B., Nolte D., Dupont D., Franklin P.A. (2025). Accounting for interspecies and intraspecies variation in swimming performance for fish passage solutions. J. Appl. Ecol..

[63] Jeremy Y., Martino J.C., Felder S., Kingsford R.T., Suthers I.M. (2026). Fish volitional and non-volitional swimming performance for fishway design: An evidence-based review. Rev. Fish Biol. Fish..

[64] Mouton A.M., Huysecom S., Buysse D., Stevens M., Van den Neucker T., Coeck J. (2014). Optimisation of adjusted barrier management to improve glass eel migration at an estuarine barrier. J. Coast. Conserv..

[65] Yeldham M.I.A., Britton J.R., Crundwell C., Davies P., Dodd J.R., Grzesiok C., Nunn A.D., Velterop R., Bolland J.D. (2025). Migration delays at head-of-tide weirs are a function of tidal cycles and river flows in anadromous twaite shad *Alosa fallax*. Estuaries Coasts.

[66] Ianniello C.F., Large S.I., Grace J.B., Hyde K.J.W., McManus M.C., Smith L.A., Deyle E.R. (2026). A multi-evidence causal investigation of water quality influences on Atlantic menhaden (*Brevoortia tyrannus*) habitat use and abundance in Narragansett Bay, USA. ICES J. Mar. Sci..

