# Supplementary material for: Seasonal Fish Passage Patterns Through an Estuarine Fishway in Relation to Tidal Hydraulics
Source: Animals (Basel). 2026 Sep 24;16(19):3014. doi: 10.3390/ani16193014 (PMC13663515; doi:10.3390/ani16193014)
Supplement: Supplementary file 1 [file animals-16-03014-s001.zip › supplementary_material.pdf]

## Supplementary Figures

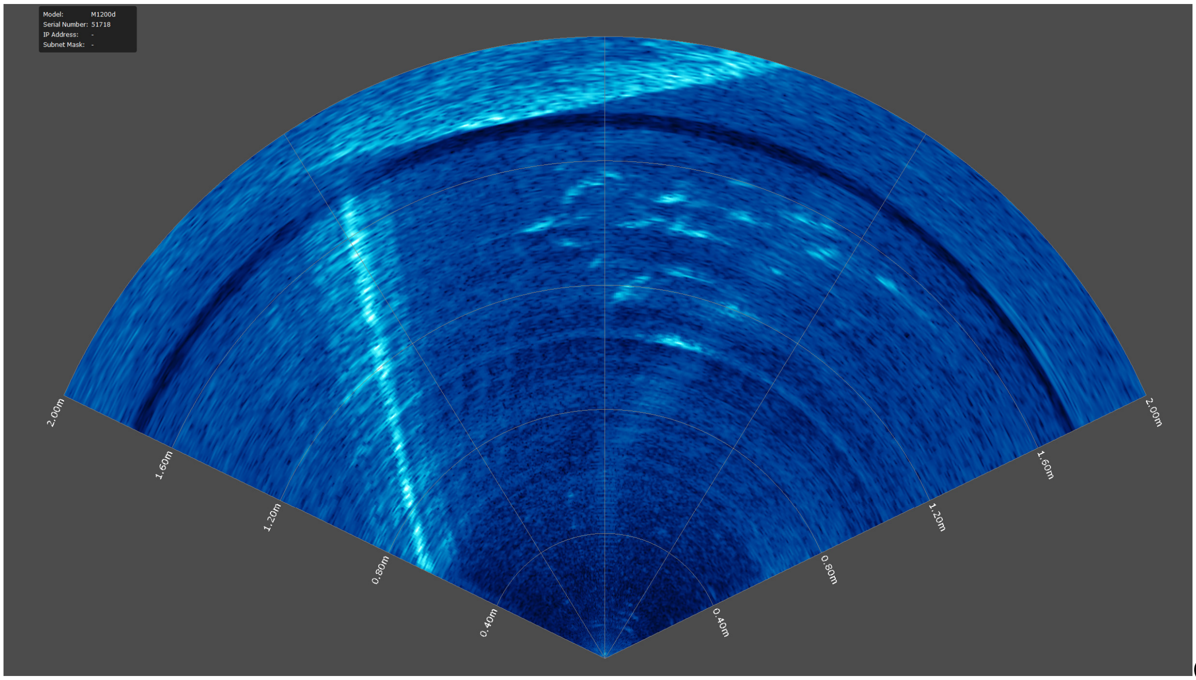


(a)

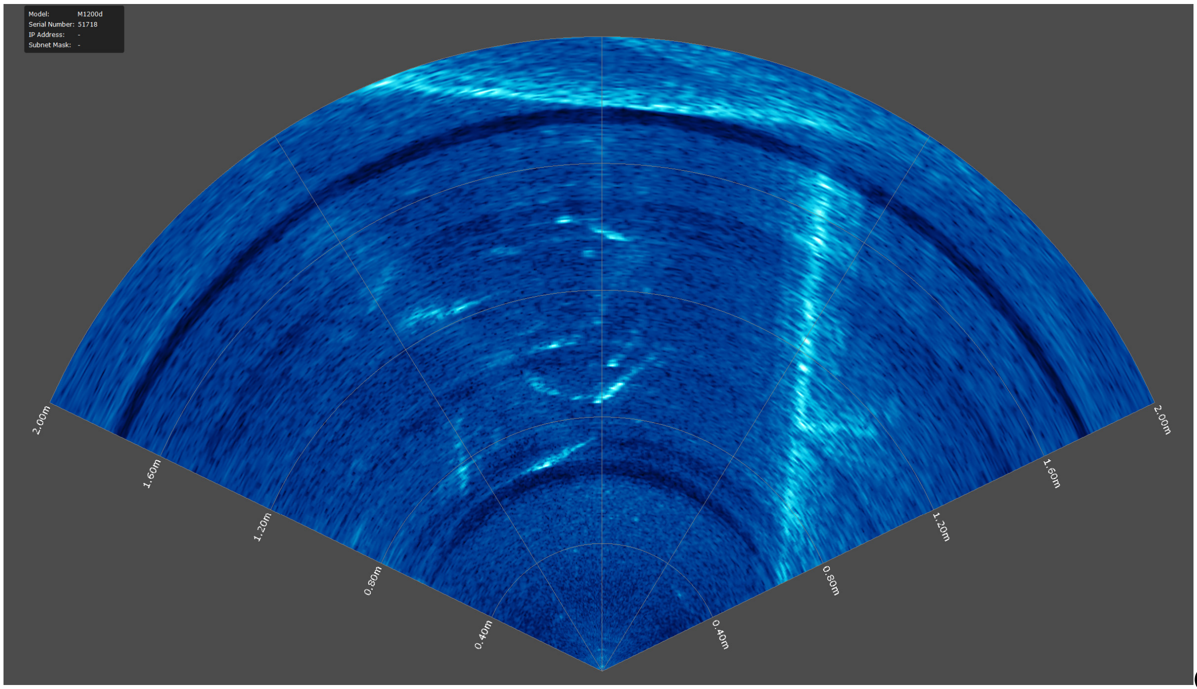


(b)

**Figure S1.** Representative sonar images recorded during (a) March–June and (b) September–November.

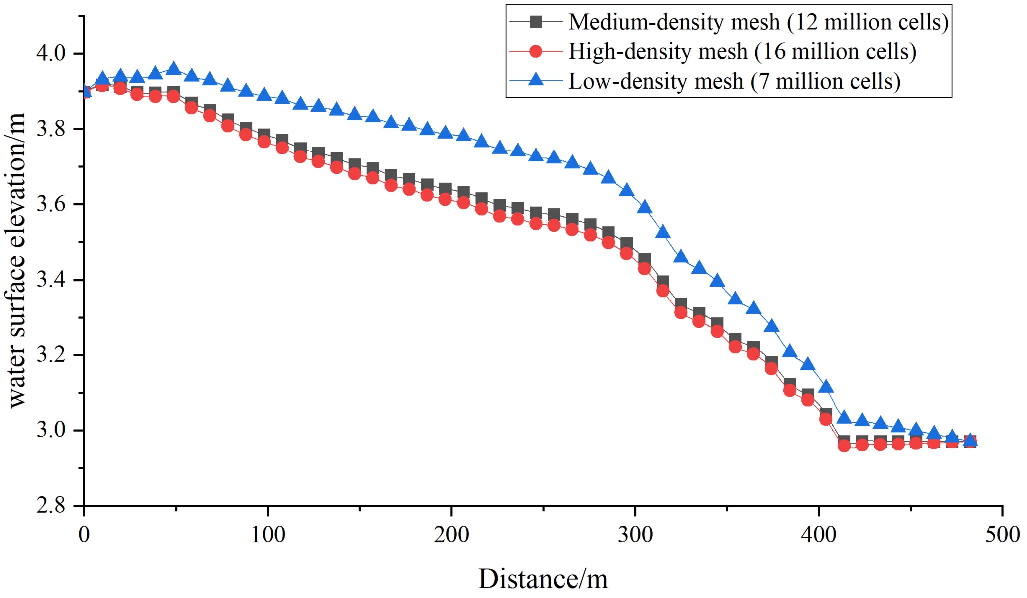


**Figure S2.** Comparison of longitudinal water-surface profiles obtained using the low-density, medium-density, and high-density meshes under a seaward tidal level of 2.97 m. The three meshes contained approximately 7 million, 12 million, and 16 million cells, respectively.

A grid-independence assessment was conducted under the representative seaward tidal level of 2.97 m, corresponding to a head difference of 0.93 m. Three structured meshes were examined, comprising approximately 7 million, 12 million, and 16 million cells, which are hereafter referred to as the low-density, medium-density, and high-density meshes, respectively. The model geometry, boundary conditions, turbulence model, and numerical settings were kept identical among the three simulations. Longitudinal water-surface profiles along the fishway centerline were extracted and compared using the result from the high-density mesh as the reference.

The three meshes produced consistent longitudinal variations in water-surface elevation (Figure S2). The mean relative difference between the medium-density and high-density meshes was 0.54%, whereas that between the low-density and high-density meshes was 3.40%. The medium-density mesh closely reproduced the water-surface profile obtained with the high-density mesh. Therefore, the medium-density mesh containing approximately 12 million cells was adopted for the numerical simulations in this study.

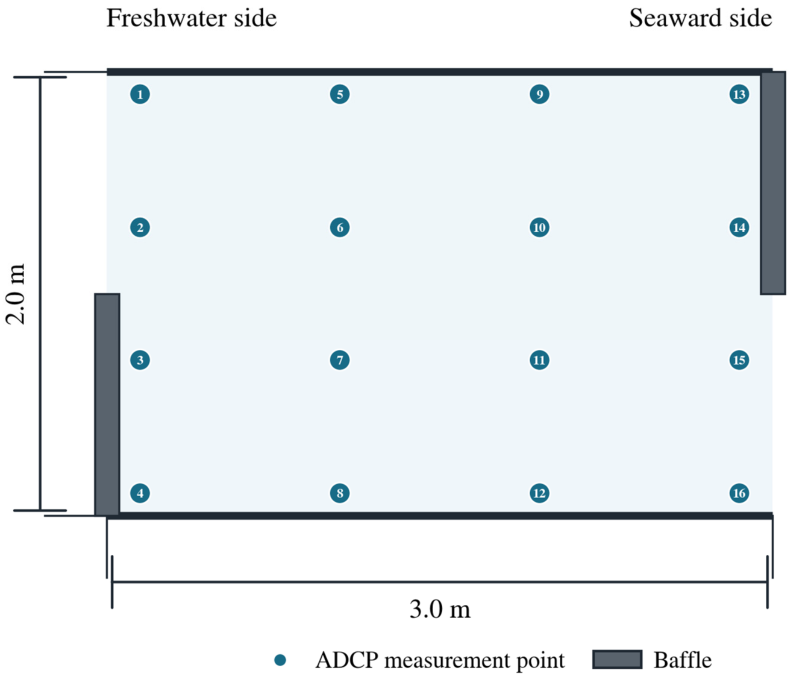


**Figure S3.** Layout of the ADCP measurement points in the representative pool. Sixteen points were arranged in four longitudinal and four transverse rows, with longitudinal and transverse spacings of approximately 1.0 and 0.6 m, respectively.

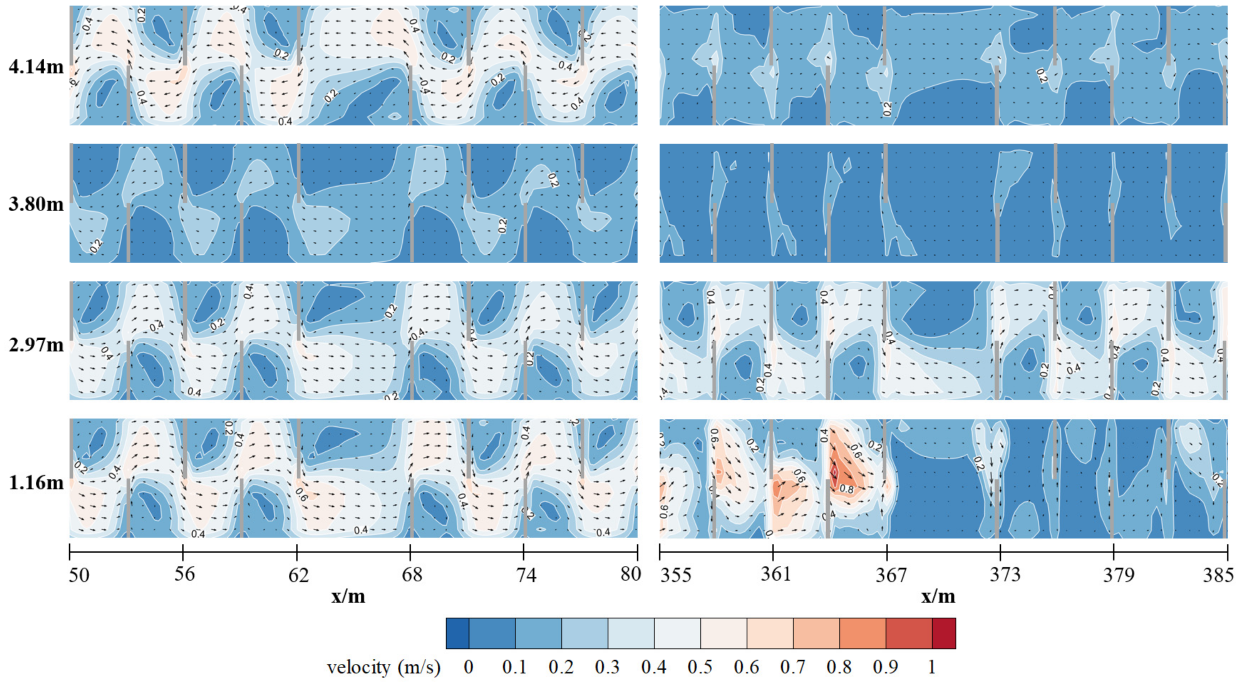


**Figure S4.** Velocity distributions with contour lines at the elevation of the notches in representative sections under different seaward tidal levels. The left column shows the freshwater-side section (z = 3.1 m), and the right column shows the seaward section (z = 2.1 m).

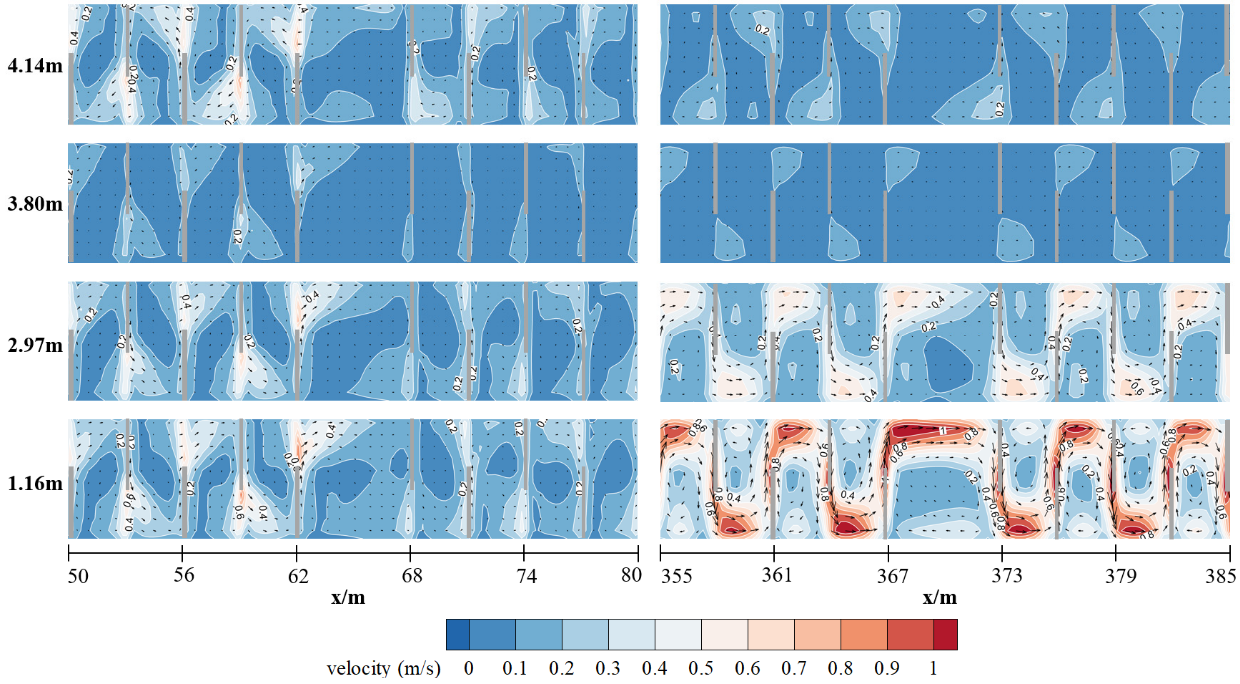


**Figure S5.** Velocity distributions with contour lines at the elevation of the orifices in representative sections under different seaward tidal levels. The left column shows the freshwater-side section (z = 2.1 m), and the right column shows the seaward section (z = 1.1 m).

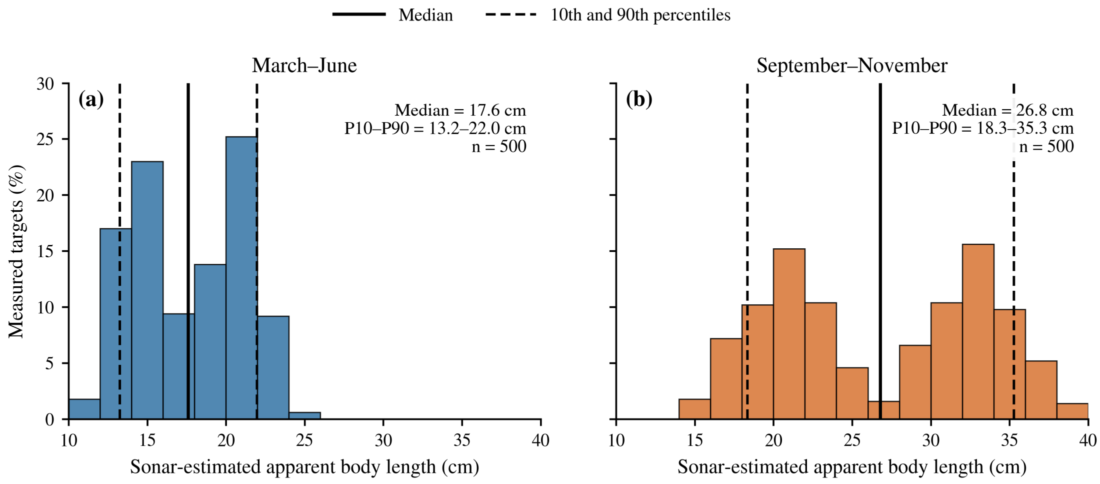


**Figure S6.** Length-frequency distributions of clarity-selected sonar targets measured during (a) March–June and (b) September–November. Histograms were constructed using common 2 cm length intervals. Solid vertical lines indicate the medians, and dashed vertical lines indicate the 10th and 90th percentiles. Each monitoring period included 500 measured targets.

# Supplementary Tables

**Table S1.** Monthly abundance and size characteristics of aquatic taxa sampled in the right-bank fishway of the Cao'e River Sluice from May to November 2017.

| Taxon | May (n) | Jun. (n) | Jul. (n) | Aug. (n) | Sep. (n) | Oct. (n) | Nov. (n) | Total (n) | Body length (cm) | Body weight (g) |
|---|---|---|---|---|---|---|---|---|---|---|
| Longarm shrimp (*Macrobrachium superbum*) | 25 | 8 | 15 | 0 | 0 | 0 | 0 | 48 | 4–9 | 1.6–9 |
| Chinese mitten crab (*Eriocheir sinensis*) | 5 | 2 | 0 | 0 | 0 | 0 | 0 | 7 | 5–6 | 4 |
| Tanshui mud crab (*Ilyoplax tansuiensis*) | 0 | 14 | 0 | 0 | 0 | 0 | 0 | 14 | 2–15 | 5–20 |
| Japanese grenadier anchovy (*Coilia ectenes*) | 632 | 163 | 654 | 0 | 4 | 9 | 3 | 1465 | 10–27 | 8–90 |
| Grass carp (*Ctenopharyngodon idellus*) | 0 | 0 | 3 | 0 | 0 | 0 | 0 | 3 | 80 | 3500 |
| Black carp (*Mylopharyngodon piceus*) | 0 | 2 | 0 | 0 | 0 | 0 | 0 | 2 | 22–32 | 100–250 |
| Blunt snout bream (*Megalobrama amblycephala*) | 0 | 0 | 3 | 2 | 1 | 0 | 1 | 7 | 23–28 | 140–400 |
| Dabry's culter (*Erythroculter dabryi*) | 0 | 2 | 0 | 0 | 0 | 0 | 0 | 2 | 40 | 400 |
| Topmouth culter (*Culter alburnus*) | 0 | 0 | 7 | 5 | 4 | 7 | 5 | 28 | 13–38 | 12–650 |
| Mongolian culter (*Culter mongolicus*) | 1 | 0 | 22 | 13 | 9 | 6 | 0 | 51 | 14–30 | 50–150 |
| Sharpbelly (*Hemiculter leucisculus*) | 574 | 107 | 211 | 0 | 0 | 0 | 0 | 892 | 8–19 | 7–90 |
| *Xenocypris davidi* | 0 | 11 | 33 | 8 | 0 | 4 | 0 | 56 | 10–30 | 30–210 |
| Silver carp (*Hypophthalmichthys molitrix*) | 0 | 0 | 6 | 16 | 18 | 12 | 0 | 52 | 15–55 | 50–2000 |
| Bighead carp (*Aristichthys nobilis*) | 0 | 0 | 14 | 10 | 18 | 12 | 8 | 62 | 15–60 | 52–2100 |
| Common carp (*Cyprinus carpio*) | 0 | 2 | 3 | 5 | 0 | 1 | 1 | 12 | 30–75 | 500–3000 |
| Crucian carp (*Carassius auratus*) | 20 | 11 | 29 | 12 | 6 | 9 | 12 | 99 | 9–25 | 25–300 |
| Yellow catfish (*Pelteobagrus fulvidraco*) | 0 | 1 | 7 | 0 | 0 | 2 | 2 | 12 | 10–22 | 14–100 |
| Japanese eel (*Anguilla japonica*) | 1 | 0 | 0 | 0 | 0 | 0 | 0 | 1 | 4 | 3 |
| Redlip mullet (*Liza haematocheila*) | 0 | 0 | 0 | 3 | 8 | 0 | 0 | 11 | 15–30 | 30–500 |
| Spotted seabass (*Lateolabrax maculatus*) | 11 | 5 | 14 | 4 | 4 | 6 | 5 | 49 | 2.5–40 | 1–1000 |
| Dark sleeper (*Odontobutis obscura*) | 0 | 0 | 1 | 0 | 0 | 0 | 0 | 1 | 20 | 50 |
| Small yellow croaker (*Larimichthys polyactis*) | 44 | 0 | 0 | 0 | 0 | 0 | 0 | 44 | 3.5–7 | 2–30 |
| Northern snakehead (*Channa argus*) | 0 | 0 | 1 | 0 | 0 | 0 | 0 | 1 | 13 | 20 |
| Spear-tail goby (*Synechogobius hasta*) | 15 | 3 | 0 | 0 | 0 | 0 | 0 | 18 | 4–10 | 2–7 |
| Obscure pufferfish (*Takifugu fasciatus*) | 1 | 1 | 0 | 0 | 0 | 0 | 0 | 2 | 16–20 | 38–42 |
| Icefish (*Hemisalanx prognathus*) | 0 | 0 | 3 | 0 | 0 | 0 | 0 | 3 | 10–12 | 10–15 |
| Halfbeak (*Hyporhamphus intermedius*) | 0 | 1 | 0 | 0 | 0 | 0 | 0 | 1 | 16 | 8 |

**Table S2.** Scenario-level hydraulic characteristics under the five simulated seaward water levels.

| Case | Boundary conditions | | Direct model outputs | | | | | | Resting-pool area below 0.3 m/s (%) | | | |
|---|---|---|---|---|---|---|---|---|---|---|---|---|
| Case | Seaward level $H_s$ (m) | Head difference $\Delta H$ (m) | Q ($m^3/s$) | $h_{min}$ (m) | $h_{mean}$ (m) | $U_{ent}$ (m/s) | Freshwater mainstream U/L range (m/s) | Seaward mainstream U/L range (m/s) | Freshwater upper z = 3.1 m | Freshwater lower z = 2.1 m | Seaward upper z = 2.1 m | Seaward lower z = 1.1 m |
| A1 | 4.14 | −0.24 | −1.37 | 3.24 | 3.48 | −0.20 | U: 0.3–0.7 (reversed)<br>L: ≈0.3–0.6 | U: ≈0.1–0.3<br>L: ≈0.1–0.3 | ≈47 | ≈97 | ≈100 | ≈100 |
| A2 | 3.80 | 0.10 | 0.41 | 2.92 | 3.15 | 0.07 | U: 0.1–0.3<br>L: 0.1–0.3 | U: 0.1–0.3<br>L: 0.1–0.3 | ≈100 | ≈99 | ≈100 | ≈100 |
| A3 | 2.97 | 0.93 | 0.60 | 2.09 | 2.31 | 0.13 | U: 0.4–0.6<br>L: 0.4–0.7 | U: 0.4–0.6<br>L: 0.4–0.7 | ≈57 | ≈91 | ≈68 | ≈76 |
| A4 | 1.16 | 2.74 | 0.31 | 0.51 | 0.55 | 0.28 | U: 0.5–0.7<br>L: 0.4–0.7 | U: 0.4–0.7 (locally 0.8–1.0)<br>L: 0.7–1.0 (locally >1.0) | ≈45 | ≈88 | ≈81 | ≈49 |
| A5 | 0.20 | 3.70 | 0.20 | 0.03 | 0.04 | 2.50 | — | — | — | — | — | — |

**Note:** $H_s$ denotes the seaward water level; $\Delta H$ is freshwater minus seaward water level; Q is the net discharge through the fishway, with negative values indicating reversed flow; $h_{min}$ and $h_{mean}$ are the minimum and mean entrance depths; and $U_{ent}$ is the section-mean entrance velocity calculated as Q divided by the wetted entrance area. U and L denote the upper and lower planes, respectively. The upper and lower velocity ranges refer to the notch- and orifice-elevation planes, respectively. Percentages are approximate plan-area fractions below 0.3 m/s in the representative resting pools. Dashes indicate that the corresponding value was not evaluated.

**Table S3.** Duration for which the entrance-depth screening criterion was met during the monitoring dates.

| **Monitoring period** | **Tidal category** | $n_d$ | ***T*a (h/cycle)** | ***P*a (%)** |
|---|---|---|---|---|
| March–June | Spring tides | 7 | 5.78 | 48.2 |
| March–June | Moderate–neap tides | 14 | 5.66 | 47.2 |
| September–November | Spring tides | 3 | 6.17 | 51.4 |
| September–November | Moderate–neap tides | 6 | 6.08 | 50.7 |

**Note:** $n_d$ denotes the number of monitoring dates; *T*a denotes the mean duration per standardized 12 h tidal cycle during which the seaward tidal level was at or above 1.16 m; and *P*a denotes the corresponding percentage of the tidal cycle. A seaward tidal level of 1.16 m was used as the water-level reference corresponding to the adopted 0.50 m entrance-depth screening criterion and scenario A4, for which the simulated minimum and mean entrance depths were approximately 0.51 and 0.55 m, respectively. This criterion was used for operational screening and was not biologically validated as a minimum depth for successful fish passage.

**Table S4.** Pearson correlations among monitoring period, tidal-stage indicators, head difference, tidal-level change rate, and daily mean air temperature at the 30 min observation level.

| Variable | Period | Late flood | Early ebb | Late ebb | ΔH | dZ/dt | Temperature |
|---|---|---|---|---|---|---|---|
| Period | 1.000 | | | | | | |
| Late flood | −0.006 | 1.000 | | | | | |
| Early ebb | 0.004 | −0.310 | 1.000 | | | | |
| Late ebb | 0.016 | −0.294 | −0.587 | 1.000 | | | |
| ΔH | −0.048 | −0.462 | −0.430 | 0.781 | 1.000 | | |
| dZ/dt | −0.028 | 0.190 | −0.460 | −0.139 | −0.002 | 1.000 | |
| Temperature | −0.185 | −0.026 | 0.015 | 0.034 | −0.051 | −0.012 | 1.000 |

**Note:** Monitoring period was coded as 0 for March–June and 1 for September–November. Tidal-stage terms were coded as binary indicators, with early flood as the reference stage. Temperature denotes daily mean air temperature.

**Table S5.** Sensitivity analysis of the negative binomial generalized linear mixed models using passage counts aggregated at a temporal resolution of 1 h.

| Factor | Contrast or increment | Upstream passage, R_adj,U (95% CI) | P | Downstream passage, R_adj,D (95% CI) | P |
|---|---|---|---|---|---|
| Monitoring period | September–November vs. March–June at early flood | 0.124 (0.053–0.291) | <0.001 | 0.096 (0.037–0.251) | <0.001 |
| Monitoring period | September–November vs. March–June at late flood | 0.100 (0.044–0.226) | <0.001 | 0.079 (0.031–0.204) | <0.001 |
| Monitoring period | September–November vs. March–June at early ebb | 0.033 (0.018–0.061) | <0.001 | 0.025 (0.011–0.058) | <0.001 |
| Monitoring period | September–November vs. March–June at late ebb | 0.114 (0.060–0.216) | <0.001 | 0.062 (0.026–0.144) | <0.001 |
| Tidal stage, March–June | Late flood vs. early flood | 1.284 (0.655–2.518) | 0.467 | 1.327 (0.759–2.319) | 0.320 |
| Tidal stage, March–June | Early ebb vs. early flood | 1.150 (0.567–2.333) | 0.699 | 1.202 (0.668–2.162) | 0.540 |
| Tidal stage, March–June | Late ebb vs. early flood | 0.492 (0.234–1.032) | 0.061 | 0.637 (0.350–1.160) | 0.140 |
| Tidal stage, September–November | Late flood vs. early flood | 1.033 (0.442–2.417) | 0.940 | 1.099 (0.531–2.275) | 0.800 |
| Tidal stage, September–November | Early ebb vs. early flood | 0.308 (0.116–0.815) | 0.018 | 0.314 (0.137–0.723) | 0.006 |
| Tidal stage, September–November | Late ebb vs. early flood | 0.452 (0.171–1.192) | 0.109 | 0.410 (0.179–0.942) | 0.036 |
| Tidal category | Spring vs. moderate–neap tides | 1.681 (0.993–2.845) | 0.053 | 1.823 (0.835–3.979) | 0.132 |
| Diel period | 06:00–12:00 vs. 00:00–06:00 | 5.929 (3.980–8.833) | <0.001 | 2.908 (2.094–4.040) | <0.001 |
| Diel period | 12:00–18:00 vs. 00:00–06:00 | 3.167 (2.170–4.623) | <0.001 | 2.691 (1.938–3.736) | <0.001 |
| Diel period | 18:00–24:00 vs. 00:00–06:00 | 1.374 (0.940–2.009) | 0.101 | 1.344 (0.965–1.872) | 0.080 |
| ΔH | Increase of 1 m | 1.026 (0.900–1.169) | 0.701 | 1.020 (0.917–1.135) | 0.716 |
| dZ/dt | Increase of 1 m/h | 1.001 (0.826–1.214) | 0.991 | 0.995 (0.847–1.168) | 0.948 |
| Temperature | Increase of 1 °C | 1.136 (1.073–1.203) | <0.001 | 1.060 (0.973–1.154) | 0.182 |

**Note:** The sensitivity analysis included 473 complete 1 h blocks from 30 monitoring dates. Passage counts and monitoring durations were summed within each block, continuous hydraulic variables were averaged, and tidal stage and diel period were assigned according to the block midpoint. Model structure, reference categories, offset, and random effect were consistent with those of the primary analysis in Table 4.

**Table S6.** Distribution of upstream passage records among tidal stages under assumed transit times.

| Period | τ (h) | EF | LF | EE | LE |
|---|---|---|---|---|---|
| March to June | 0.0 | 4,759 (10.0) | 10,100 (21.2) | 25,541 (53.7) | 7,200 (15.1) |
| | 0.5 | 8,587 (18.0) | 7,313 (15.4) | 24,580 (51.6) | 7,120 (15.0) |
| | 1.0 | 10,268 (21.6) | 6,611 (13.9) | 22,808 (47.9) | 7,913 (16.6) |
| | 1.5 | 9,879 (20.8) | 7,143 (15.0) | 20,530 (43.1) | 10,048 (21.1) |
| | 2.0 | 7,193 (15.1) | 8,281 (17.4) | 18,754 (39.4) | 13,372 (28.1) |
| | 2.5 | 6,922 (14.5) | 8,887 (18.7) | 16,218 (34.1) | 15,573 (32.7) |
| | 3.0 | 7,302 (15.3) | 8,905 (18.7) | 14,106 (29.6) | 17,287 (36.3) |
| September to November | 0.0 | 1,206 (23.3) | 608 (11.7) | 509 (9.8) | 2,864 (55.2) |
| | 0.5 | 1,130 (21.8) | 282 (5.4) | 614 (11.8) | 3,161 (60.9) |
| | 1.0 | 793 (15.3) | 174 (3.4) | 748 (14.4) | 3,472 (66.9) |
| | 1.5 | 601 (11.6) | 107 (2.1) | 959 (18.5) | 3,520 (67.9) |
| | 2.0 | 281 (5.4) | 117 (2.3) | 1,128 (21.7) | 3,661 (70.6) |
| | 2.5 | 182 (3.5) | 126 (2.4) | 1,368 (26.4) | 3,511 (67.7) |
| | 3.0 | 129 (2.5) | 154 (3.0) | 1,760 (33.9) | 3,144 (60.6) |

**Notes:** $\tau$ denotes the assumed transit time, and $\tau = 0$ represents the recorded time of crossing at the exit monitoring section. EF, early flood; LF, late flood; EE, early ebb; LE, late ebb. Entries are upstream passage counts, with percentages of the corresponding monitoring-period total in parentheses. Observation midpoints were shifted backward by $\tau$ hours along the corresponding tidal time series, and tidal stages were reassigned using the surrounding tidal turning points. Counts were retained at their original 30 min resolution. The full dataset comprised 529 intervals covering 264.5 h during March–June and 432 intervals covering 216 h during September–November. Three autumn intervals with zero upstream counts were excluded because the preceding tidal turning points required for the lag analysis were unavailable. A common set of 529 and 429 intervals was therefore used across all lag scenarios for the two periods, containing 47600 and 5187 upstream passage records, respectively. Percentages may not sum to 100 because of rounding.

**Table S7.** Spatial similarity of upstream passage intensity distributions under assumed transit times.

| **τ (h)** | $r_{s,\mathrm{M}}$ | $r_{s,\mathrm{S}}$ | $r_{s,\mathrm{obs,M}}$ | $r_{s,\mathrm{obs,S}}$ |
|---|---|---|---|---|
| 0.0 | 1.000 | 1.000 | 1.000 | 1.000 |
| 0.5 | 0.666 | 0.767 | 0.768 | 0.752 |
| 1.0 | 0.477 | 0.621 | 0.603 | 0.589 |
| 1.5 | 0.178 | 0.507 | 0.361 | 0.470 |
| 2.0 | 0.078 | 0.402 | 0.245 | 0.391 |
| 2.5 | −0.008 | 0.399 | 0.138 | 0.376 |
| 3.0 | −0.051 | 0.344 | 0.083 | 0.350 |

**Notes:** τ denotes the assumed transit time, and τ = 0 represents the recorded time of crossing at the exit monitoring section. M and S denote March–June and September–November, respectively. The coefficients $r_s$ describe Spearman correlations between the 240 grid values at each assumed lag and the corresponding zero-lag distribution. The subscript obs indicates correlations calculated only for cells containing observations in both distributions. Observation midpoints were shifted backward by τ hours, and hydraulic conditions were rematched using the tidal data retained for the original Figure 10 analysis. Grid limits and spacing were unchanged. Observed cell means were retained, and empty cells were filled by inverse distance weighting using the eight nearest observed cells and a distance exponent of two. The full analytical dataset comprised 529 intervals covering 264.5 h during March–June and 432 intervals covering 216 h during September–November. For comparison across all lag scenarios using a common set of observations, 18 autumn intervals with zero upstream counts were excluded because the preceding tidal data required for hydraulic rematching were incomplete for at least one lag. Table S7 therefore includes 529 and 414 intervals, respectively. The corresponding upstream passage totals were 47,600 and 5,187. Correlations are descriptive measures of spatial similarity.
